# REK1 positively regulates salt stress by phosphorylating RBOHD to modulate ROS homeostasis in Arabidopsis

**DOI:** 10.1111/tpj.71093

**Published:** 2026-08-19

**Authors:** Ao Li, Shuo Wang, Xiushuo Liang, Sheng Luo, Honghong Hao, Renjing Wan, Shaowu Xue

**Affiliations:** ^1^ Hubei Hongshan Laboratory College of Life Science and Technology, Huazhong Agricultural University 430070 Wuhan China; ^2^ School of Life Sciences, Institute for Systems Biology, Jianghan University 430056 Wuhan China

**Keywords:** salt stress, seed germination, reactive oxygen species, phosphorylation, *Arabidopsis thaliana*

## Abstract

Salinity induces the accumulation of reactive oxygen species (ROS) in plants, thereby regulating plant growth and development, including seed germination. However, the molecular mechanisms underlying the regulation of ROS homeostasis in response to salt stress remain largely unknown. Here, we showed that an leucine‐rich‐repeat receptor‐like kinase, REK1, regulates ROS production by phosphorylating the NADPH oxidase respiratory burst oxidase homolog D (RBOHD) in Arabidopsis in response to salt stress. Salt‐induced ROS accumulates extensively in *rek1* mutants, causing stress that reduces the seed germination rate of *rek1* below that of the wild‐type (Col‐0), resulting in the salt‐sensitive phenotype. Furthermore, REK1 interacts with RBOHD and phosphorylates it at Ser163, thereby regulating its NADPH oxidase activity and influencing ROS production under salt stress. Genetic data revealed that the salt‐sensitive phenotype of *rek1* is rescued in *rek1 rbohd* mutants. In summary, our study revealed a salt stress regulation mechanism that modulates ROS production in Arabidopsis through the REK1‐RBOHD pathway.

## Significance Statement

The study shows that REK1, a member of the leucine‐rich repeat receptor‐like kinase family, increases plant tolerance to salt stress during seed germination by phosphorylating NADPH oxidase respiratory burst oxidase homolog D, thereby regulating its oxidase activity and reactive oxygen species levels.

## INTRODUCTION

Seed germination is a significant stage in the plant life cycle, during which seeds are particularly susceptible to environmental stresses, including salinity (Luo et al., 2020; Penfield, 2017). High salinity causes ion toxicity, osmotic stress, and secondary stress in plants, further disrupting the physiological balance within plants, leading to reduced seed germination, impaired plant growth, and abnormal development (Ismail et al., 2014; Slama et al., 2015; Yang & Guo, 2018; Zhang et al., 2021; Zhu, 2002). Furthermore, reactive oxygen species (ROS) play a key but largely uncharacterized role in inhibiting seed germination under salt stress (Luo et al., 2020; Ortiz‐Espín et al., 2017).

ROS regulate diverse plant growth, development, and stress response processes, and they are highly reactive oxygen‐containing molecules produced during plant cell metabolism, including superoxide anions (O^2−^), hydrogen peroxide (H_2_O_2_), hydroxyl radicals (OH·), and singlet oxygen (^1^O_2_) (Gill & Tuteja, 2010; Mittler, 2017; Smirnoff & Arnaud, 2018; Waszczak et al., 2018).

ROS produced by NADPH oxidases RESPIRATORY BURST OXIDASE HOMOLOG (RBOH), such as RBOHD, have dual roles in plant stress responses (Suzuki et al., 2011; Torres et al., 2002). For instance, loss of RBOHD function can lead to increased cadmium influx and enhanced toxicity (Hafsi et al., 2022); in the *rbohd* mutant, mild hypoxic stress was not observed to mitigate the deleterious effects of salinity on the growth of wild‐type plants or to induce a pre‐adaptive response in the root system (Wang et al., 2019), demonstrating that RBOHD plays a protective role under certain stress conditions. However, excessive ROS accumulation can cause oxidative damage to cellular components and inhibit seed germination as well as plant growth and development (Mittler, 2006; Ugalde et al., 2021; Xie et al., 2011). For example, the transcription of *RBOHD* is activated under salt stress (Luo et al., 2020; Ma et al., 2012), and in *rbohd*, ROS toxicity is reduced, and the seed germination rate is higher than that of Col‐0 under salt treatment (Luo et al., 2020). In addition, growing research has shown that multiple protein kinases are capable of phosphorylating the N‐ and C‐terminal domains of RBOHD and regulating its oxidase activity, thereby influencing ROS production and responses to external stress (Ding et al., 2024; Dubiella et al., 2013; Kimura et al., 2012, 2020; Li et al., 2014).

Receptor‐like kinases (RLKs) are a large family of protein kinases that respond to various stresses and transmit signals from the cell membrane to multiple components of the cytoplasm, thereby regulating plant growth, development, and stress response processes (Cargnello & Roux, 2011; Matsuzaki et al., 2010; McCarty & Chory, 2000; Shiu & Bleecker, 2001; Zhang et al., 2021; Zhao et al., 2018). Leucine‐rich‐repeat receptor‐like kinase (LRR‐RLK) is the largest subfamily of RLKs, with approximately 230 members identified in Arabidopsis (Cui et al., 2022; Soltabayeva et al., 2022; Yin et al., 2024). Typical LRR‐RLKs include FLS2, which participates in PAMP‐triggered immunity, BRI1, which is engaged in BR signaling, and BAK1, which functions as a coreceptor (Gómez‐Gómez & Boller, 2000; Li et al., 2014; Sun et al., 2013; Yuan et al., 2021). Specifically, RLK7, an LRR‐RLK, positively regulates salt tolerance in Arabidopsis seedlings via MPK3/6 signaling cascade (Zhou et al., 2022). In maize, the LRR‐RLK protein ZmMIK2 suppresses drought‐ and salt stress responses by stabilizing ZmC2DP1 through phosphorylation and buffering Ca^2+^ signals (Yang et al., 2025). Furthermore, OsSTLK, a member of the LRR‐RLK family in rice, positively regulates salt stress tolerance (Lin et al., 2020). However, the detailed mechanism underlying the LRR‐RLK‐mediated salt stress response remains largely unexplored.

RBOHD‐dependent ROS generation is a typical downstream event of RLK activation under various stresses (Ding et al., 2024; Ma et al., 2025; Shiu & Bleecker, 2001). For example, an LRR‐RLK ALR1 phosphorylates RBOHD, triggering a burst of ROS, which in turn inhibits RAE1‐mediated STOP1 degradation, activates organic acid secretion to detoxify aluminum ions, and improves aluminum tolerance (Ding et al., 2024). CYSTEINE‐RICH RLK2 (CRK2) positively regulates the microbial‐associated molecular pattern‐triggered ROS burst and plant innate immunity by phosphorylating the C‐terminus of RBOHD (Kimura et al., 2020). However, whether the RLK‐RBOHD module functions in plant salt tolerance remains unknown.

In this study, we identified a salt‐sensitivity mutant *rek1*, that exhibited reduced seed germination under salinity conditions. *REK1* (*ROOT ELONGATION RECEPTOR KINASES*) encodes an *LRR‐RLK*, which was previously described to regulate plant development by controlling downstream gene expression, thereby participating in the balance between stress resistance and growth (Ogawa‐Ohnishi et al., 2022; Wang et al., 2022). We showed here that REK1 positively regulates redox signaling under salt treatment. Further characterization demonstrated that REK1 physically interacts with RBOHD and phosphorylates its N‐terminus at Ser163, thereby inhibiting its oxidase activity. Consequently, the *rek1 rbohd* double mutant exhibited salt tolerance phenotypes similar to those of the *rbohd* mutant, indicating that RBOHD is genetically epistatic to REK1 under salt stress. Taken together, our findings reveal a novel component that alleviates salinity‐induced ROS toxicity during seed germination and provide a new REK1‐RBOHD module in salt tolerance.

## RESULTS

### *rek1* is sensitive to salt stress

**Figure 1:**
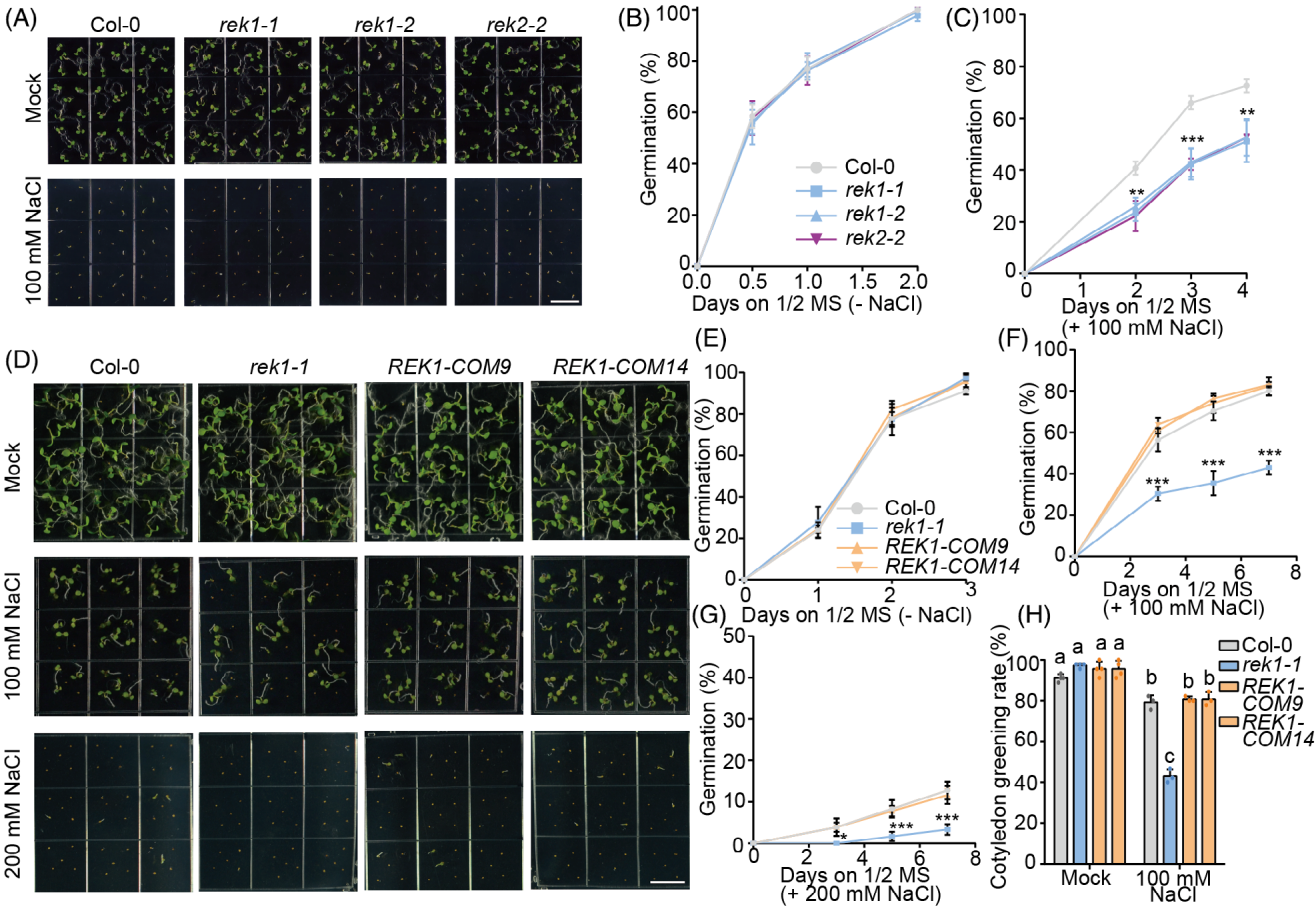
REK1 positively regulates salt stress. (A) Seed germination of Col‐0, *rek1‐1*, *rek1‐2*, and *rek2‐2* on 1/2 MS medium with or without 100 mM NaCl treatment. Bar, 1 cm. (B, C) The quantitative analysis of the germination rates is shown for control (B) and 100 mM NaCl (C) treatment. Percentages are the average of three repeats ± SD (*n* = 45). Asterisks indicate a significant difference (**P < 0.01; ***P < 0.001) according to one‐way anova with Tukey's test. (D) Seed germination of Col‐0, *rek1‐1*, *REK1‐COM9*, and *REK1‐COM14* on 1/2 MS medium with or without exogenous NaCl treatment (100 and 200 mM). Bar, 1 cm. (E–G) The quantitative analysis of the germination rates is shown for control (E), 100 mM NaCl (F), and 200 mM NaCl (G) treatments. Percentages are the average of three repeats ± SD (*n* = 45). Asterisks indicate a significant difference (*P < 0.05; ***P < 0.001) according to one‐way anova with Tukey's test. (H) The statistics of the cotyledon greening rate on the seventh day of 100 mM NaCl treatment. Significant differences were determined via two‐way anova with Tukey's test; different lowercase letters indicate significant differences (*P* < 0.05).

To search for new factors involved in salt stress, we performed salt stress assays on a library of T‐DNA insertion mutants of RLKs in Arabidopsis. Among these, the *rek1‐1*, *rek1‐2*, and *rek2‐2* mutants showed increased sensitivity to salt stress in seed germination (Figure 1A). These mutants carry T‐DNA insertions in the coding sequence (CDS) region of genes encoding LRR‐RLK (Figure S1a,b). The PCR assay confirmed the T‐DNA insertion in these three mutants (Figure S1c–e). The seed germination phenotypes were tested at 100 mM NaCl (Figure 1A). On 1/2 MS medium, all the genotypes tested showed comparable germination rates (Figure 1A,B); however, the germination rates of *rek1‐1*, *rek1‐2*, and *rek2‐2* seeds were lower than that of the wild‐type (Columbia‐0, Col‐0) under 100 mM NaCl stress (Figure 1A,C). These results suggest that both *rek1* and *rek2* are sensitive to salt stress, prompting us to select *REK1* for subsequent studies.

To test whether the salt stress‐sensitive phenotype of *rek1* is indeed caused by loss of *REK1* function, we attempted to restore this phenotype by introducing complementary transgenic lines (*REK1‐COM‐9* and *REK1‐COM14*) that express the *REK1* coding region under the native *REK1* promoter. The quantitative reverse transcription‐PCR (qRT‐PCR) analysis showed that *rek1‐1* and *rek1‐2* were knockdown mutants (Figure S1f). Moreover, the transcript levels of *REK1* in *COM9* and *COM14* were similar to those in Col‐0 (Figure S1g). In addition, *REK1* was overexpressed in Col‐0 plants (*REK1‐OE4* and *REK1‐OE11*) driven by the constitutive CaMV35S promoter (Figure S2a). The seed germination phenotypes were evaluated in the presence of NaCl at concentrations of 100 and 200 mM (Figure 1D). *rek1* also exhibited a significantly lower cotyledon greening rate than Col‐0 after 100 mM NaCl treatment (Figure 1H). Salt stress hypersensitivity of *rek1* was rescued in the complementation lines, as evidenced by seed germination rate and cotyledon greening rate comparable to those of Col‐0 (Figure 1D–H). Moreover, the seed germination rate and cotyledon greening rate (Figure S2b–f) of the *REK1‐OE* lines under salt stress were also similar to those of Col‐0. These suggest that the salt‐sensitive phenotype of *rek1* is indeed due to the loss of the *REK1* gene.

### The localization of *REK1* and its expression patterns

**Figure 2:**
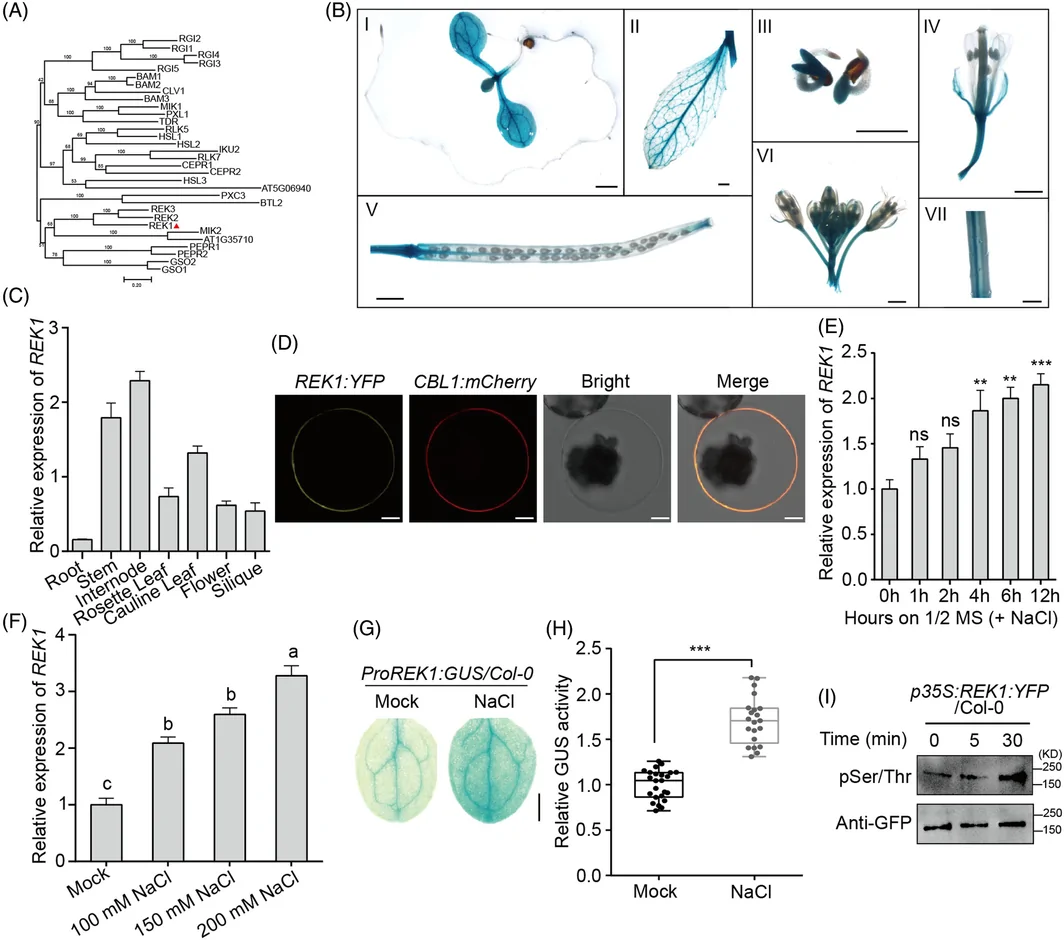
The expression patterns of REK1 and its induction by salt treatment. (A) The phylogenetic tree of Arabidopsis leucine‐rich‐repeat receptor‐like kinase (LRR‐RLK) subfamily XI members. (B) GUS staining of *ProREK1:GUS/Col‐0* transgenic line. Arabidopsis seedlings (I), cauline leaves (II), germinating seeds (III), floral sepals (IV), siliques (V), floral organs (VI), and stems (VII). Bars, 1 mm. (C) Quantitative reverse transcription‐PCR (qRT‐PCR) analysis of *REK1* tissue expression in root, stem, internode, rosette leaf, cauline leaf, flower, and silique of Arabidopsis. Values represent mean ± SD (*n* = 3). (D) Localization of REK1 in Arabidopsis protoplasts. REK1‐YFP and PM marker CBL1‐mCherry constructs were simultaneously transformed into Arabidopsis protoplasts. Bars, 10 μm. (E) The expression levels of *REK1* were analyzed by qRT‐PCR after treating 5‐day‐old Col‐0 seedlings with 150 mM NaCl in 1/2 MS medium for 0, 1, 2, 4, 6, and 12 h. The mean ± SD data were obtained from three independent experiments for each treatment group (*n* = 3). Asterisks indicate a significant difference (ns, no significance; **P < 0.01; ***P < 0.001) according to one‐way anova with Tukey's test. (F) qRT‐PCR analysis of *REK1* expression in 5‐day‐old Col‐0 seedlings grown in 1/2MS media with 0, 100, 150, and 200 mM NaCl for 12 h. Significant differences were determined via one‐way anova with Tukey's test; different lowercase letters indicate significant differences (*P* < 0.05). Mean ± SD values were obtained from three independent experiments for each treatment (*n* = 3). (G) GUS staining of 5‐day‐old seedlings treated with or without 150 mM NaCl for 12 h. NaCl treatment enhances *REK1* expression *in vivo*. Representative images of Arabidopsis leaves are shown. Bars, 0.5 mm. (H) Quantitative analysis of β‐glucuronidase (GUS) activity as shown in (G). The experiments were performed in three biological replicates, and a similar trend was observed across all replicates. Asterisks indicate a significant difference (***P < 0.001) according to Student's *t*‐test. The average percentages of three repeats ± SD are shown (*n* ≥ 21). (I) Seven‐day‐old *Pro35S:REK1:YFP* transgenic seedlings were subjected to 150 mM NaCl for 0, 5, and 30 min. REK1‐YFP protein was enriched with anti‐GFP beads. Anti‐GFP and anti‐pSer/Thr antibodies were used to detect total REK1 and phosphorylated REK1, respectively.

To elucidate its genetic basis, we constructed a phylogenetic tree including all members of the Arabidopsis LRR‐RLK subfamily XI (Zhang et al., 2026). As shown in Figure 2A, this tree provides a comprehensive view of the evolutionary relationships within this subfamily. *REK1*, also known as PSYR1, negatively regulates root development and positively regulates stress response (Ogawa‐Ohnishi et al., 2022; Wang et al., 2022). Another two genes (*REK2/PSYR2* and *REK3/PSYR3*) are located on the same branch as *REK1*. We further obtained the T‐DNA insertion mutant *rek3‐3* and subjected it to salt stress treatment (Figure S3a–d). Under normal conditions, no significant differences in seed germination rates were observed among these genotypes (Figure S3b,c). Under salt stress, *rek1* and *rek2* exhibited a lower germination rate than that of Col‐0. However, *rek3* displayed germination rates similar to those of Col‐0 (Figure S3b,d).

To further explore how *REK1* regulates the salt stress response, its expression patterns and subcellular localization were examined. β‐glucuronidase (GUS) staining of the *ProREK1:GUS* transgenic lines showed that *REK1* was abundantly expressed in leaves at various developmental stages and was also expressed in stems and calyx (Figure 2B). Consistent with the GUS staining results, qRT‐PCR showed that *REK1* was primarily expressed in leaves and stems, with lower expression levels in roots (Figure 2C). To investigate the subcellular localization of REK1, we constructed the *Pro35S:REK1‐YFP* vector and transiently transformed it into the Arabidopsis protoplasts. Confocal microscopy revealed that the yellow fluorescence for REK1 overlapped with the red fluorescence of the plasma membrane marker CBL1, demonstrating that REK1 is localized to the plasma membrane (Figure 2D).

To investigate how REK1 responds to salt stress, qRT‐PCR assay showed that salt stress increased the expression of *REK1* (Figure 2E). Subsequently, *REK1* expression was affected by stress severity, as indicated by the fact that more *REK1* transcripts were induced by 200 mM NaCl than by 100 mM NaCl (Figure 2F). To further study stress‐induced *REK1* expression, we determined that REK1 expression is strongly elicited by salt stress using *ProREK1:GUS/Col‐0* transgenic Arabidopsis (Figure 2G,H). In addition, we examined the kinase activity of REK1 in response to salt treatment. We treated the 7‐day‐old *REK1* overexpression transgenic seedlings with 150 mM NaCl for 0, 5, or 30 min, respectively. After enriching the proteins with GFP beads, we detected kinase activity using phosphorylation‐specific antibodies. As shown in Figure 2I, a 30‐min salt treatment significantly enhanced the autophosphorylation activity of the kinase REK1 compared with untreated controls. This result indicates that salt stress directly promotes the intrinsic kinase activity of REK1.

### *REK1* might affect redox signaling during plant salt stress

**Figure 3:**
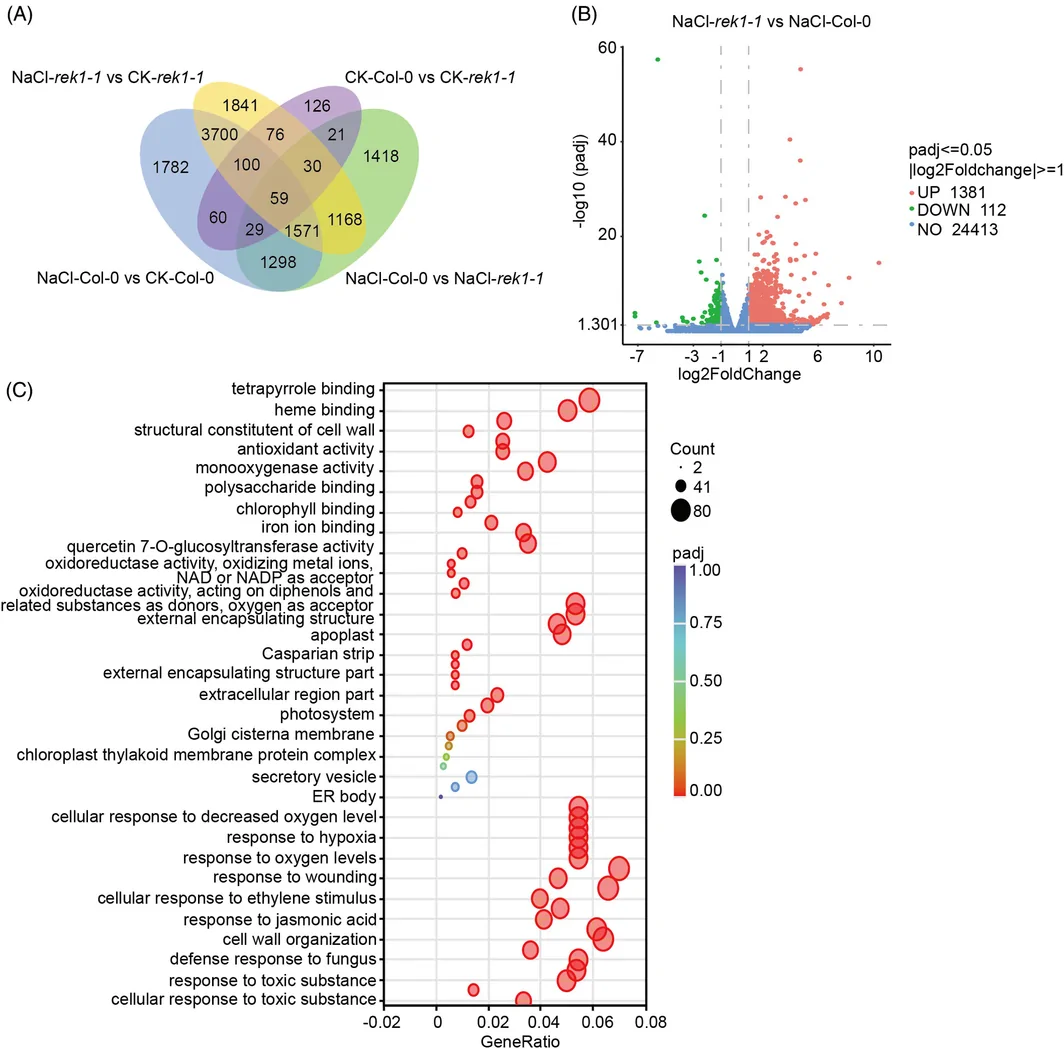
RNA sequencing analysis of *rek1‐1* and Col‐0 under salt treatment. (A) Venn diagram showing the overlap of differentially expressed genes among CK‐Col‐0 versus CK‐*rek1‐1*, NaCl‐Col‐0 versus NaCl‐*rek1‐1*, NaCl‐Col‐0 versus CK‐Col‐0, and NaCl‐*rek1‐1* versus NaCl‐*rek1‐1* under NaCl treatment (|log2 fold change| ≥ 1, *P* < 0.05). (B) Volcano plots showing differentially expressed genes (DEGs) (|log2 fold change| ≥ 1, *P* < 0.05) between *rek1‐1* and Col‐0 plants under salt stress. The *REK1*‐regulated genes were identified, comprising 112 downregulated and 1381 upregulated genes in *rek1‐1* relative to Col‐0 plants. (C) GO analysis of biological pathways enriched in *REK1*‐regulated genes under salt stress.

To further clarify the function of *REK1* in salt stress signaling, we analyzed the transcriptome in Col‐0 and *rek1‐1* mutants under salt stress. Five‐day‐old seedlings were treated with or without 150 mM NaCl, and then total RNA was extracted from whole seedlings for RNA sequencing (RNA‐seq) analysis. Using |log2 fold change| ≥1 and *P* < 0.05 as thresholds, we identified different numbers of differentially expressed genes (DEGs) in various groups of Col‐0 and *rek1‐1* mutants with or without NaCl treatment, respectively (Figure 3A). The volcano plot shows that under non‐salt‐treated conditions, *rek1‐1* had a relatively small number of DEGs compared with Col‐0 (Figure S4a). We identified 1381 upregulated and 112 downregulated genes in NaCl‐*rek1‐1* compared with NaCl‐Col‐0, respectively (Figure 3B). We therefore performed gene ontology enrichment analysis on these DEGs. The analysis showed that the DEGs were significantly enriched for oxidative stress, redox pathways, and oxygen levels (Figure 3C).

To gain insight into the transcriptional network downstream of the REK1‐ROS signaling module, we analyzed the RNA‐seq dataset to identify differentially expressed transcription factors (TFs) in *rek1‐1* relative to Col‐0 under salt stress. Among the TF‐encoding DEGs, we selected several candidates (*RAB18*, *MYB3*, *bZIP9*, *WRKY25*, *WRKY46*, and *bZIP53*) with documented roles in ROS homeostasis or stress responses for qRT‐PCR validation (Doll et al., 2020; Hao et al., 2024; Lijuan et al., 2024; Ortiz‐Espín et al., 2017; Zhang et al., 2001; Zhu et al., 2023). These qRT‐PCR results were consistent with the RNA‐seq data, confirming the robustness of the transcriptomic analysis (Figure S4b–g). Collectively, these findings indicate that the REK1 module differentially regulates distinct subsets of stress‐responsive TFs under salt stress. Moreover, qRT‐PCR results showed that exogenous application of H_2_O_2_ induced *REK1* expression (Figure S5a). Methyl viologen (MV) acts as an oxidative stress inducer and can produce ROS in plants (Cui et al., 2019; Kalinina et al., 2024). The expression level of *REK1* was also increased by exogenous application of MV (Figure S5b). Collectively, these RNA‐seq data provide evidence supporting REK1 as a positive regulator of redox signaling at the global gene‐expression level.

### REK1 affects ROS production under salt stress

**Figure 4:**
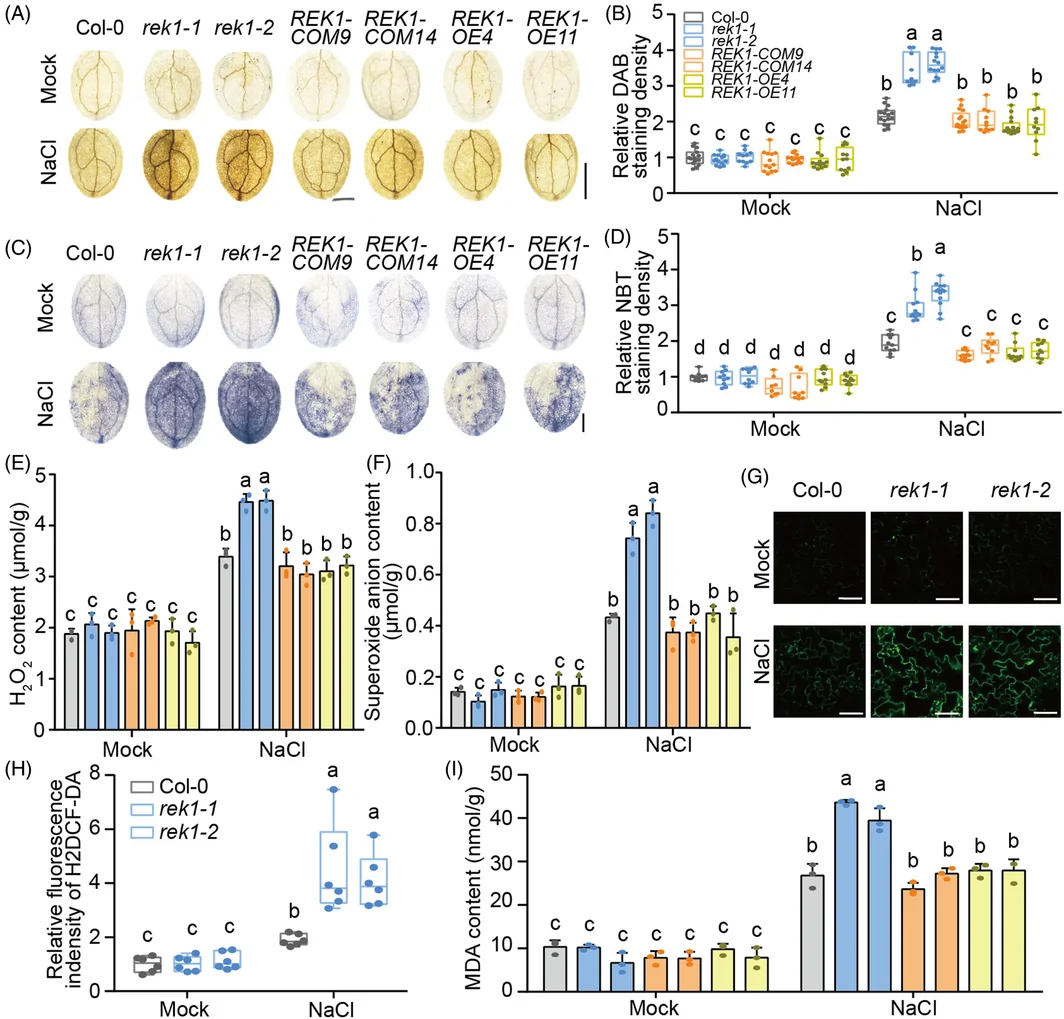
Excessive accumulation of reactive oxygen species (ROS) in *rek1* under salt stress. (A, B) Four‐day‐old leaves were used for 3,3′‐diaminobenzenamine (DAB) staining (A), and staining was counted relatively quantitatively (B). Bars, 1 mm. Significant differences were determined via two‐way anova with Tukey's test; different lowercase letters indicate significant differences (*P* < 0.05). Mean ± SD values were obtained from three independent experiments for each treatment (*n* ≥ 10). (C, D) Four‐day‐old leaves were selected for nitro blue tetrazolium chloride (NBT) staining (C), and the staining intensity was quantitatively analyzed (D). Bars, 0.5 mm. Significant differences were determined via two‐way anova with Tukey's test; different lowercase letters indicate significant differences (*P* < 0.05). Mean ± SD values were obtained from three independent experiments for each treatment (*n* ≥ 8). (E, F) H_2_O_2_ (E) and O^2−^ (F) contents in plants of 5‐day‐old Col‐0, *rek1*, *REK1‐COM*, and *REK1‐OE* seedlings with and without 150 mM NaCl treatment. Significant differences were determined via two‐way anova with Tukey's test; different lowercase letters indicate significant differences (*P* < 0.05). Mean ± SD values were obtained from three independent experiments for each treatment (*n* = 3). (G) Seven‐day‐old Col‐0, *rek1‐1*, and *rek1‐2* seedlings were treated with 50 mM NaCl for 0 and 30 min, followed by staining with H2DCF‐DA. Fluorescent signals were taken using a confocal microscope (Leica SP8). Bars, 60 μm. (H) The relative H2DCF‐DA fluorescence signals in Col‐0, *rek1‐1*, and *rek1‐2* lines were determined based on confocal images using ImageJ software. Values are means ± SD (*n* = 6). Different letters above the error bars indicate significant differences at *P* < 0.05, as determined by two‐way anova with Tukey's test. (I) Malondialdehyde (MDA) contents in plants of 5‐day‐old Col‐0, *rek1*, *REK1‐COM*, and *REK1‐OE* seedlings with and without 150 mM NaCl treatment. Significant differences were determined via two‐way anova with Tukey's test; different lowercase letters indicate significant differences (*P* < 0.05). Mean ± SD values were obtained from three independent experiments for each treatment (*n* = 3).

Oxidative stress induced by salt stress is primarily due to the excessive production and accumulation of ROS in plants (Kimura et al., 2020; Kwak et al., 2003; Sagi & Fluhr, 2006). To examine the potential association between REK1 function and ROS, we performed assays to measure two primary ROS, H_2_O_2_ and O^2−^. We examined H_2_O_2_ and O^2−^ levels in 5‐day‐old seedlings of Col‐0, *rek1*, *REK1‐COM*, and *REK1‐OE* lines with or without 150 mM NaCl treatment using 3,3′‐diaminobenzenamine (DAB) (Figure 4A,B) and nitro blue tetrazolium chloride (NBT) (Figure 4C,D) staining, respectively. There were no differences in H_2_O_2_ and O^2−^ levels between genotypes under non‐NaCl conditions, whereas H_2_O_2_ and O^2−^ accumulation in the leaves of *rek1* mutant were much higher than that of Col‐0 after exogenous salt stress treatments (Figure 4A–D). In comparison, the *REK1‐COM* and *REK1‐OE* lines showed ROS staining levels comparable to those of Col‐0. The quantitative assay results (Figure 4E,F) were in accordance with the staining evidence (Figure 4A–D). To more sensitively and quantitatively assess ROS levels, we performed H2DCF‐DA fluorescence imaging on leaves treated with salt for 0 and 30 min. Consistent with the above results, *rek1‐1* and *rek1‐2* displayed stronger fluorescence intensity than Col‐0 under salt stress (Figure 4G,H), confirming that loss of REK1 leads to enhanced ROS production.

Excessive accumulation of ROS causes oxidative damage to plants (Sagi & Fluhr, 2006; Waszczak et al., 2018). Malondialdehyde (MDA) is a by‐product of oxidative stress in plants and is commonly used as an indicator to assess cell membrane integrity and damage (Gill & Tuteja, 2010). To elucidate the relationship between REK1‐mediated changes in ROS and seed germination, we measured MDA content in the aforementioned plants. Under non‐saline stress conditions, MDA levels showed no significant differences among the tested plants (Figure 4I). Conversely, MDA levels in *rek1* were significantly higher than those in Col‐0 under saline stress. In comparison, MDA levels in *REK1‐COM/OE* transgenic lines were comparable to those in Col‐0 (Figure 4I).

Collectively, ROS accumulated in the *rek1* mutant under salt stress, which induces oxidative stress in plants and damages cell membrane integrity. This may be one factor contributing to the salt sensitivity of the *rek1* mutant. Thus, REK1 may affect the ROS‐related signaling pathways in response to salt stress.

### REK1 interacts with RBOHD

**Figure 5:**
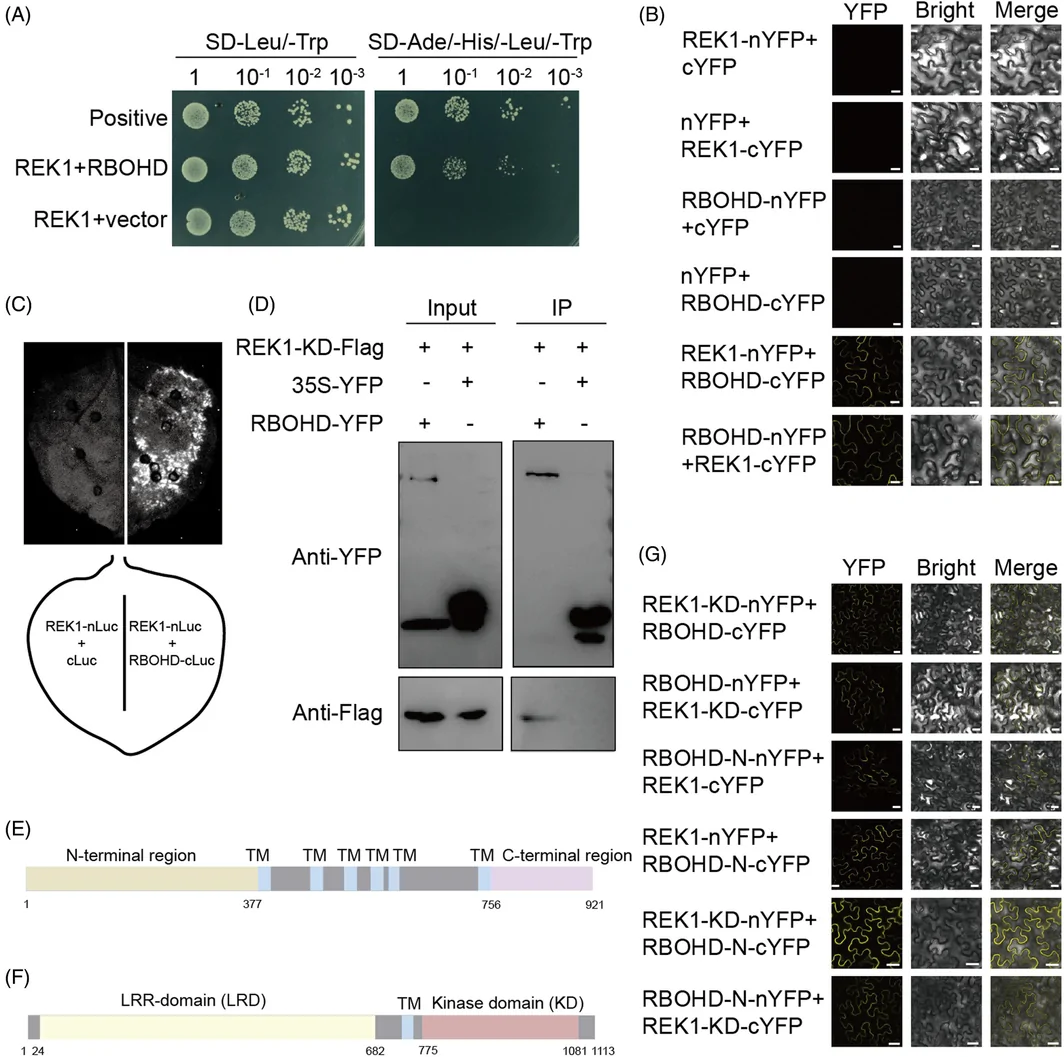
REK1 interacts with respiratory burst oxidase homolog D (RBOHD). (A) Yeast two‐hybrid assay showing interaction between REK1 and RBOHD. The coding sequences for the REK1 kinase domain (KD) and RBOHD were cloned into the pBT3‐STE vector (Cub) and pPR3‐N vector (Nub), respectively. Different plasmid combinations were transformed into the yeast strain NMY51. After culturing cells on SD‐Leu‐Trp medium for 2 days, protein interactions were detected using SD‐Ade‐His‐Leu‐Trp medium. Positive control: pCCW‐Alg5 and PAI‐Alg5 were combined for transformation. (B) Bimolecular fluorescence complementation (BiFC) assay showing the interaction of REK1 with RBOHD. Bars, 20 μm. (C) Luciferase complementation imaging (LCI) assay showing that REK1 interacts with RBOHD in tobacco leaves. Tobacco leaves were co‐transformed with constructs REK1‐nLUC and RBOHD‐cLUC, and luciferase signals were captured after 3 days of *Agrobacterium* infiltration. Combinations of REK1‐nLUC and Vector‐cLUC were used as a negative control. (D) *In vivo* co‐immunoprecipitation (Co‐IP) assay showing the interaction between the REK1 kinase domain and RBOHD. Constructs encoding FLAG‐tagged REK1 and YFP‐tagged RBOHD were transiently co‐transfected into Col‐0 Arabidopsis protoplasts and immunoprecipitated using anti‐YFP affinity magnetic beads. (E) Schematic representation of RBOHD structure. N‐terminal region (RBOHD‐N, AAs 1–377); C‐terminal region (RBOHD‐C, AAs 756–921). (F) Schematic representation of REK1 structure. LRR‐domain (REK1‐LRD, AAs 1–682); Kinase domain (REK1‐KD, AAs 775–1113). (G) BiFC assays showing that the kinase domain of REK1 interacts with the N‐terminus of RBOHD. Bars, 30 μm.

To explore the relationship between REK1 and ROS in response to salt stress and to further elucidate the mechanism by which REK1 mediates salt stress, we used a yeast two‐hybrid (Y2H) system to screen for ROS‐related genes and identify potential REK1 interactors. The Y2H result displayed that RBOHD interacts with REK1 (Figure 5A). We next used the bimolecular fluorescence complementation (BiFC) assay to further investigate the interaction between REK1 and RBOHD. The simultaneous transient expression of REK1 and RBOHD in the leaves of *Nicotiana benthamiana* resulted in YFP fluorescence at the plasma membrane. In contrast, no fluorescence signal was observed when REK1 or RBOHD was expressed alone (Figure 5B). This suggests that REK1 interacts with RBOHD at the plasma membrane. Subsequently, to confirm the interaction between REK1 and RBOHD, we performed luciferase complementation imaging (LCI) assays in *N. benthamiana* and detected robust fluorescence signals (Figure 5C). The results from Arabidopsis protoplast co‐immunoprecipitation (Co‐IP) experiments showed that the kinase domain of REK1 could immunoprecipitate with RBOHD‐YFP, but not with YFP (Figure 5D). These results collectively demonstrate the interaction between these two proteins. To further probe which regions of REK1 and RBOHD are required for the interaction, a series of truncated fragments for the two proteins was generated, and their interaction was validated using the BiFC system (Figure 5E,F). The BiFC results showed that REK1 interacts with the N‐terminus of RBOHD (RBOHD‐N) through its kinase domain (REK1‐KD) (Figure 5G). However, no YFP fluorescence was observed when REK1 was co‐expressed with the C‐terminus of RBOHD (RBOHD‐C), as was the case when RBOHD was co‐expressed with the LRR‐domain of REK1 (REK1‐LRD), suggesting that no interaction between these combinations occurred (Figure S6). Therefore, these results indicate that the kinase domain of REK1 primarily interacts with the N‐terminus of RBOHD.

### REK1 phosphorylates RBOHD and inhibits its activity

**Figure 6:**
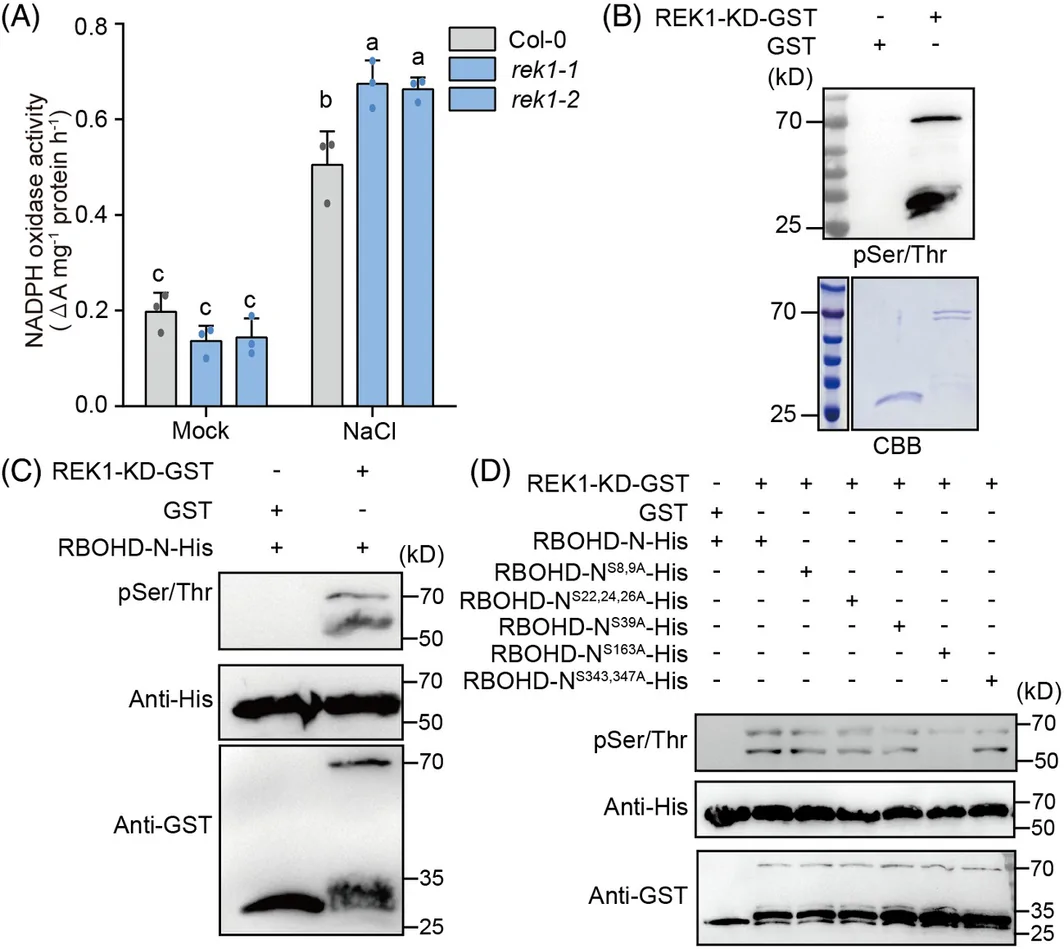
REK1 phosphorylates respiratory burst oxidase homolog D (RBOHD) and inhibits its oxidase activity. (A) NADPH oxidase activity in control and 150 mM NaCl‐treated seedlings. Significant differences were analyzed by two‐way anova with Tukey's test; different lowercase letters indicate significant differences (*P* < 0.05). Mean ± SD values were obtained from three independent experiments (*n* = 3). (B) *In vitro* kinase assay for REK1 kinase domain (REK1‐KD). CBB indicates Coomassie blue staining. (C) *In vitro* kinase assay showing phosphorylation of RBOHD‐N by REK1‐KD. Recombinant RBOHD‐N‐His and REK1‐KD‐GST proteins were incubated in protein kinase buffer containing ATP. Phosphorylated RBOHD‐N‐His and REK1‐KD‐GST were detected by anti‐pSer/Thr after gel electrophoresis (top panel), recombinant RBOHD‐N‐His protein was detected by anti‐His antibody (middle panel), and recombinant REK1‐KD‐GST protein was detected by anti‐GST antibody (bottom panel). (D) Phosphorylation of RBOHD‐N and its mutant forms by REK1‐KD detected *in vitro* with pSer/Thr antibody.

Salt stress activates RBOHD, driving ROS production and accumulation in plants (Luo et al., 2020; Zhang et al., 2021; Zhao et al., 2020). Since REK1 functions in response to salt stress and interacts with RBOHD, we further tested whether REK1 affects RBOHD oxidase activity under salt stress. The results revealed that NADPH oxidase activity did not differ among genotypes under normal growth conditions (Figure 6A; Figure S7a). In contrast, NADPH oxidase activity was activated in *rek1* after salt treatment (Figure 6A), but did not differ from Col‐0 in *COM* and *OE* lines of REK1 (Figure S7a), suggesting that REK1 negatively regulates the NADPH oxidase activity of RBOHD.

REK1 acts as a protein kinase, primarily phosphorylating target proteins, thereby affecting a range of downstream signaling responses (Soltabayeva et al., 2022). Therefore, we next examined whether REK1 can phosphorylate RBOHD. We expressed the recombinant proteins REK1‐KD‐GST and RBOHD‐N‐His *in vitro*. The phosphorylation activity of REK1‐KD was detected *in vitro* (Figure 6B). Then, we found that the REK1 kinase domain could phosphorylate the N‐terminus of RBOHD in an *in vitro* kinase assay (Figure 6C). To identify REK1‐targeted phosphorylation sites, we selected several serine (S) residues (S8/9, S22/24/26, S39, S163, S343/347) at the N‐terminus of RBOHD (Figure S7d) as candidates by combining website prediction analysis (https://phosphat.uni‐hohenheim.de/) with known phosphorylation sites of RBOHD (Castro et al., 2021; Kadota et al., 2015; Wang et al., 2020). We purified RBOHD‐N‐His recombinant proteins corresponding to different S sites mutated to Alanine (A) for *in vitro* kinase assays (Figure 6D). In the presence of REK1‐KD‐GST, the phosphorylation of RBOHD‐N^S163A^ was lower than that of intact RBOHD‐N‐His, suggesting that S163 is the major site recognized by REK1 (Figure 6D).

**Figure 7:**
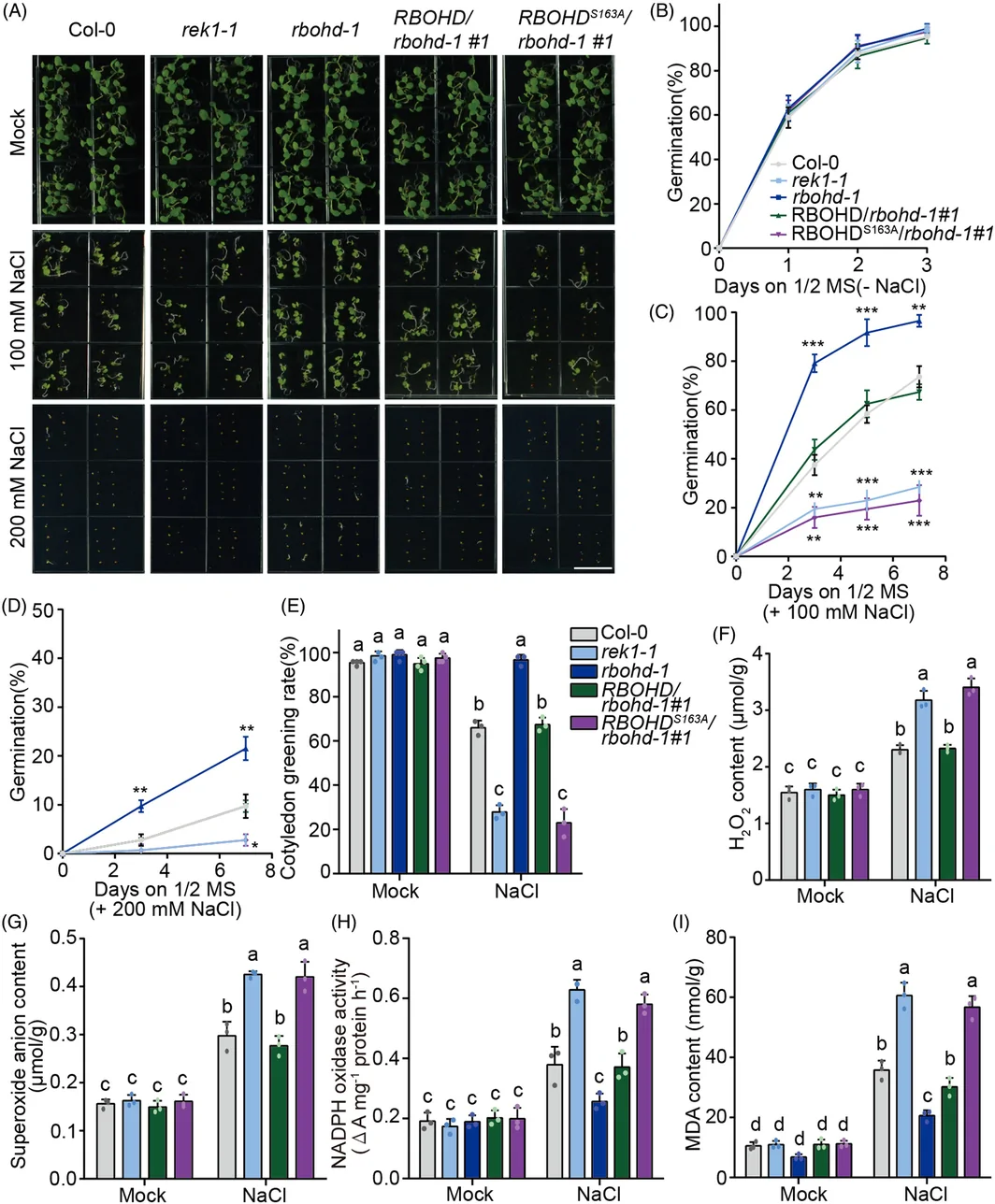
REK1‐mediated phosphorylation of respiratory burst oxidase homolog D (RBOHD) negatively regulates reactive oxygen species (ROS) production and salt stress tolerance. (A) Seed germination of Col‐0, *rek1‐1*, *rbohd‐1*, *RBOHD/rbohd‐1#1*, *RBOHD* ^*S163A*^ */rbohd‐1#1* on 1/2 MS medium with or without NaCl treatment (100 and 200 mM). The same batch of seeds was analyzed. Bar, 1 cm. (B–D) The quantitative analysis of the germination rates is shown for control (B), 100 mM NaCl (C), and 200 mM NaCl (D) treatments. Percentages are the average of three repeats ± SD (*n* = 48). Asterisks indicate a significant difference (*P < 0.05; **P < 0.01; ***P < 0.001) according to one‐way anova with Tukey's test. (E) Statistics for the cotyledon greening rate on the seventh day of 100 mM NaCl treatment. Percentages are the average of three repeats ± SD (*n* ≥ 3). Significant differences were determined via two‐way anova with Tukey's test; different lowercase letters indicate significant differences (*P* < 0.05). (F, G) H_2_O_2_ (F) and O^2−^ (G) contents in plants of 5‐day‐old Col‐0, *rek1‐1*, *RBOHD/rbohd‐1#1*, *RBOHD* ^*S163A*^ */rbohd‐1#1* seedlings with and without 150 mM NaCl treatment. Significant differences were determined via two‐way anova with Tukey's test; different lowercase letters indicate significant differences (*P* < 0.05). Mean ± SD values were obtained from three independent experiments for each treatment (*n* = 3). (H) NADPH oxidase activity in control and 150 mM NaCl‐treated seedlings. Significant differences were analyzed by two‐way anova with Tukey's test; different lowercase letters indicate significant differences (*P* < 0.05). Mean ± SD values were obtained from three independent experiments (*n* = 3). (I) Malondialdehyde (MDA) content in plants of 5‐day‐old Col‐0, *rek1‐1*, *RBOHD/rbohd‐1#1*, *RBOHD* ^*S163A*^ */rbohd‐1#1* seedlings with and without 150 mM NaCl treatment. Significant differences were determined via two‐way anova with Tukey's test; different lowercase letters indicate significant differences (*P* < 0.05). Mean ± SD values were obtained from three independent experiments for each treatment (*n* = 3).

To determine the biological significance of RBOHD phosphorylation under salt stress, we obtained the *rbohd* mutant (SALK_109396) and showed that RBOHD was barely expressed in *rbohd‐1* through PCR and qRT‐PCR assays (Figure S7b,c). Then we acquired transgenic plants by transforming *rbohd‐1* with *ProRBOHD:RBOHD* and *ProRBOHD:RBOHD* ^*S163A*^ vectors, respectively (Figure S7e). We phenotyped these lines under normal growth conditions and under salt stress (100 and 200 mM NaCl), and observed no differences in growth among all genotypes under normal conditions (Figure 7A,B). While the seed germination rates and cotyledon greening rate of *RBOHD* ^*S163A*^ */rbohd‐1#1* and *rek1‐1* were lower than those of Col‐0 under salt stress, the seed germination rates and cotyledon greening rate did not differ between *RBOHD/rbohd‐1#1* and Col‐0 (Figure 7A,C–E). Correspondingly, the H_2_O_2_ and O^2−^ contents, NADPH oxidase activity, and MDA content in *RBOHD* ^*S163A*^ */rbohd‐1#1* were also higher than those in Col‐0 upon salt stress, as was the case in rek1‐1 (Figure 7F–I), which confirmed that the inhibition of REK1 on RBOHD is correlated to the phosphate site S163. Taken together, REK1 phosphorylates RBOHD on S163 and suppresses its ROS production activity.

### RBOHD functions downstream of REK1 in salt stress response

**Figure 8:**
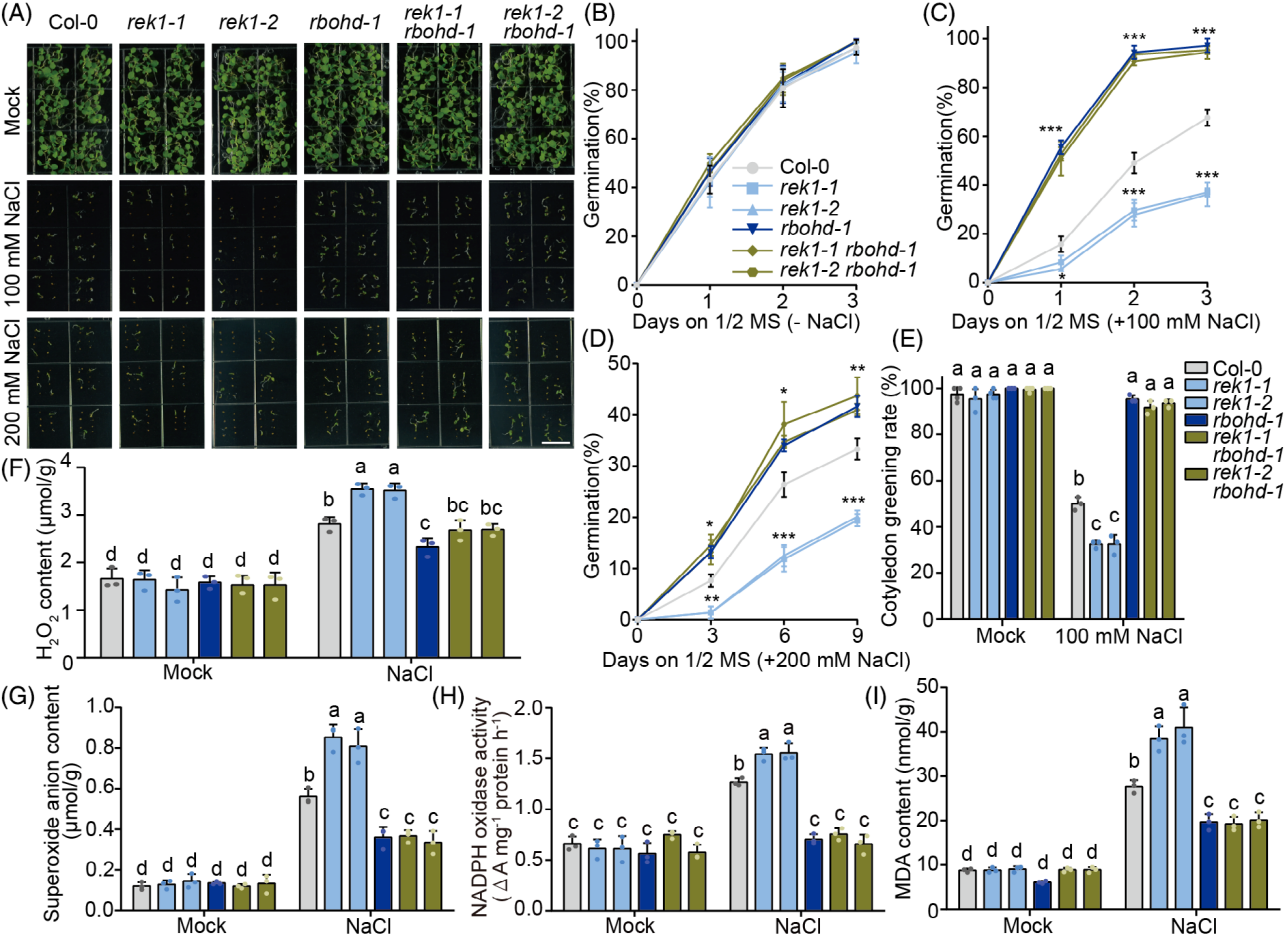
REK1 functions upstream of respiratory burst oxidase homolog D (RBOHD) under salt stress. (A) Seed germination of Col‐0, *rek1*, *rbohd*, and *rek1 rbohd* on 1/2 MS medium with or without NaCl treatment (100 and 200 mM). The same batch of seeds was analyzed. Bar, 1 cm. (B–D) The quantitative analysis of the germination rates is shown for control (B), 100 mM NaCl (C), and 200 mM NaCl (D) treatments. Percentages are the average of three repeats ± SD (*n* ≥ 36). Asterisks indicate a significant difference (*P < 0.05; **P < 0.01; ***P < 0.001) according to one‐way anova with Tukey's test. (E) Statistics of cotyledon greening rate on the seventh day of the 100 mM NaCl treatment. Percentages are the average of three repeats ± SD (*n* ≥ 3). Significant differences were determined via one‐way anova with Tukey's test; different lowercase letters indicate significant differences (*P* < 0.05). (F, G) H_2_O_2_ (F) and O^2−^ (G) in plants of 5‐day‐old Col‐0, *rek1*, and *rek1 rbohd* seedlings with and without 150 mM NaCl treatment. Significant differences were determined via two‐way anova with Tukey's test; different lowercase letters indicate significant differences (*P* < 0.05). Mean ± SD values were obtained from three independent experiments (*n* = 3). (H) NADPH oxidase activity in control and 150 mM NaCl‐treated seedlings. Significant differences were analyzed by two‐way anova with Tukey's test, and different lowercase letters indicate significant differences (*P* < 0.05). Mean ± SD values were obtained from three independent experiments (*n* = 3). (I) Malondialdehyde (MDA) content in plants of 5‐day‐old Col‐0, *rek1*, and *rek1 rbohd* seedlings with and without 150 mM NaCl treatment. Significant differences were determined via two‐way anova with Tukey's test; different lowercase letters indicate significant differences (*P* < 0.05). Mean ± SD values were obtained from three independent experiments for each treatment (*n* = 3).

To further determine the genetic relationship between REK1 and RBOHD in response to salt stress, we crossed *rek1‐1* and *rek1‐2* with *rbohd‐1* to obtain two double mutants, respectively (Figure S8a–d). Under salt stress, the seed germination rate and cotyledon greening rate of *rbohd‐1* were higher than those of Col‐0 (Figure 8A–E), indicating that *rbohd‐1* was more tolerant to salt stress. The *rek1* mutant is sensitive to salt stress, whereas the *rek1 rbohd* double mutant exhibits a phenotype consistent with *rbohd‐1* (Figure 8A–E), suggesting that RBOHD acts downstream of REK1 in responses to salt stress.

Meanwhile, the results of DAB and NBT experiments showed that under salt stress, the H_2_O_2_ and O^2−^ contents in the *rek1 rbohd* double mutant were lower than those in Col‐0, as in the *rbohd* (Figure S9a–d). Quantitative measurements of ROS content are consistent with the above results (Figure 8F,G). Moreover, NADPH oxidase activity in *rek1 rbohd* was lower than that in Col‐0 upon salt treatment, as it was in *rbohd* (Figure 8H). Correspondingly, the MDA content in *rek1 rbohd* was lower than that in Col‐0 under salt stress conditions, as was the case with *rbohd* (Figure 8I). As a control, there was no difference in NADPH oxidase activity and MDA content among all genotypes in the absence of salt treatment (Figure 8H,I). Overall, REK1 functions upstream of RBOHD, thereby affecting RBOHD oxidase activity and ROS production under salt stress.

## DISCUSSION

Throughout the plant life cycle, RLKs play crucial roles in growth and development as well as in response to external stresses (Cui et al., 2022). As the largest subfamily of RLK, LRR‐RLKs regulate diverse biological processes and have been well studied in plant growth and development, as well as responses to biotic stresses (Ding et al., 2024; Gómez‐Gómez & Boller, 2000; Soltabayeva et al., 2022; Xun et al., 2020). However, their involvement in regulating abiotic stresses, particularly salt stress, remains largely unknown. Acting through negative regulation of cell wall remodeling genes and activation of stress response TFs during ligand deficiency, REK1/PSYR1 of the LRR‐RLK XI subfamily has been reported to regulate the trade‐off between root development and stress resistance (Ogawa‐Ohnishi et al., 2022; Wang et al., 2022). However, the role of REK1 under abiotic stress and its underlying molecular mechanisms remain largely unexplored. Here, our research showed that the *rek1* mutant exhibited reduced seed germination under salt stress (Figure 1), displaying a salt‐sensitive phenotype and indicating that REK1 positively regulates the seed germination response to salt stress. In addition, to determine whether the germination phenotypes of *rek1* and *rbohd* mutants under salt stress were caused by ionic toxicity or osmotic stress, we assessed seed germination under conditions of 150 mM KCl and 250 mM mannitol. Under both conditions, no significant differences in germination rates were observed among the Col‐0, *rek1*, *rbohd*, and *rek1 rbohd* mutants (Figure S10). These results demonstrate that the REK1‐RBOHD module specifically promotes germination under NaCl stress rather than mediating general ionic or osmotic stress responses during germination.

Previous studies have demonstrated that the NADPH oxidase RBOHD regulates multiple physiological processes in Arabidopsis, particularly in response to biotic stresses (Kadota et al., 2015; Ma et al., 2012; Mittler et al., 2022). For instance, RBOHD can be phosphorylated by CRK2, thereby modulating flg22‐induced ROS production and defense responses against pathogens (Kimura et al., 2020). The BAK1‐BIK1 receptor kinase complex responds to biotic stress by generating ROS via RBOHD activation (Chen et al., 2025). Additionally, RBOHD can be phosphorylated by CPK16 and ALR1, respectively, to regulate hypoxic and aluminum stress signaling pathways in Arabidopsis (Ding et al., 2024; Yu et al., 2024). However, the role of RBOHD in salt stress signaling pathways remains largely unknown. In our study, RNA‐seq and ROS content measurements indicate that REK1 regulates redox signaling under salt stress (Figures 3 and 4). Further experimental results demonstrate that REK1 can interact with the NADPH oxidase RBOHD and phosphorylate it (Figures 5 and 6C). Our study links REK1 function to RBOHD, a well‐known hub of stress signaling. This enhances understanding of the RLKs‐RBOHD‐mediated salt stress signaling response.

RBOHD is a plasma membrane‐localized NADPH oxidase harboring six transmembrane domains (Kwak et al., 2003; Ma et al., 2012). Previous studies have reported that both the N‐ and C‐termini of RBOHD are regulated by phosphorylation by distinct kinases, which influence ROS production; most prior reports indicate that these kinases promote RBOHD‐mediated ROS generation (Wang et al., 2020). However, PBL13 can phosphorylate RBOHD, thereby negatively regulating ROS production (Lee et al., 2020). Additionally, OsCPK4‐mediated phosphorylation of OsRBOHB in rice negatively regulates ROS production (Tao et al., 2025). Therefore, it is crucial to elucidate the molecular mechanisms underlying the negative regulation of RBOHD under abiotic stress. Under salt stress, elevated cytosolic Ca^2+^ activates RBOHD via direct EF‐hand binding and phosphorylation by Ca^2+^‐dependent protein kinases, forming a positive feedback loop that amplifies ROS production (Demidchik et al., 2018). This amplification mechanism is important for robust stress signaling, but also carries the risk of uncontrolled ROS accumulation. In our study, we found that REK1 directly interacts with RBOHD and phosphorylates its N‐terminal S163 site (Figures 5 and 6), which has been reported to negatively regulate ROS production (Castro et al., 2021), thereby suppressing its oxidase activity and reducing ROS production (Figure 7). The mutation of RBOHD S163 to Ala elevates its NADPH oxidase activity (Figure 7H), leading to increased ROS accumulation (Figure 7F,G) and reduced seed germination rate under salt stress (Figure 7A–E), indicating REK1 as a critical negative regulator that phosphorylates RBOHD at Ser163, thereby limiting RBOHD activity and preventing excessive ROS buildup. Moreover, the RBOHD protein may be subject to both positive and negative regulation by different kinases, and the REK1‐RBOHD module may therefore function as a ‘safety valve’ that counterbalances Ca^2+^‐driven RBOHD activation, ensuring precise control of ROS signaling during the salt stress response. Thus, the positive and negative feedback loops operate in concert to shape ROS signals with appropriate intensity and duration, enabling effective stress adaptation without incurring oxidative damage.

The physiological relevance of the REK1‐RBOHD regulatory module is further supported by our observations that *rek1* mutants exhibit enhanced ROS accumulation, oxidative damage, and impaired germination under salt stress, all of which are largely rescued by *rbohd* mutation. These results highlight the biological importance of the REK1‐mediated negative feedback loop in preventing ROS overproduction during salt stress. Additionally, we found that although *REK1‐OE* exhibited higher kinase activity under salt stress than *REK1‐COM* (Figure S11), it did not show a further reduction in RBOHD oxidase activity, ROS levels, or an enhanced seed germination rate (Figure 4; Figures S2 and S7a). Therefore, there may be other regulatory factors or unknown pathways within plants that prevent the excessive suppression of ROS. Such mechanisms might involve salt‐induced protein phosphatases that dephosphorylate RBOHD, compensatory activation of RBOHD‐activating kinases, enhanced turnover of hyperphosphorylated RBOHD, or other pathways. Future studies aimed at identifying the molecular components of this regulation will be critical for a complete understanding of ROS homeostasis under salt stress.

To summarize, we propose the following working model to elucidate the function of REK1 in the salt stress response of Arabidopsis. In Col‐0, REK1 expression and kinase activity are induced upon salt stress (Figure 2E–I). It negatively regulates RBOHD's oxidase activity by interacting with RBOHD and phosphorylating its N‐terminal S163 residue (Figures 5 and 6), thereby ensuring that ROS remain within a moderate, beneficial range and maintaining normal germination. In *rek1*, however, the absence of REK1 removes the inhibition of RBOHD, leading to excessive activation of RBOHD and the production of deleterious ROS (Figure 4), ultimately reducing seed germination rates (Figure 1). Our research reveals a novel, fine‐tuned salt stress response mechanism mediated by REK1 that directly regulates RBOHD activity through protein interactions and phosphorylation. This deepens our understanding of how plants precisely balance ROS levels during seed germination under salt stress.

## MATERIALS AND METHODS

### Plant materials and growth conditions

*Arabidopsis thaliana* Col‐0 (wild‐type) and T‐DNA insertion mutants used in this study were obtained from the Arabidopsis Biological Resource Center (ABRC, Columbus, OH, USA http://www.arabidopsis.org). The *rek1‐1 rbohd‐1* and *rek1‐2 rbohd‐1* mutants were generated by crossing *rek1‐1* or *rek1‐2* with *rbohd‐1*, respectively.

Surface‐sterilized seeds were sown on half‐strength Murashige and Skoog (MS) agar (1/2 MS) medium containing 1% (w/v) sucrose and stratified at 4°C in the dark for 2 days. Plants were then grown in a glasshouse at 21°C, with a 16‐h‐light/8‐h‐dark cycle with 80 μmol m^−2^ sec^−1^ light intensity.

### Generation of transgenic plants

For the complementation assay of *rek1‐1* and *rek1‐2*, the *REK1* promoter region was amplified using the primer pair REK1‐COM‐F/REK1‐COM‐R, cloned into the pGreen0179 vector, and the vector was transformed into *rek1‐1* and *rek1‐2*, respectively, to obtain complementary plants using *Agrobacterium*‐mediated inflorescence transformation (Xu & Xu, 2018). To obtain *REK1* overexpression plants, the primer pair REK1‐OE‐F/REK1‐OE‐R was used to amplify the full‐length CDS of *REK1*, which was then recombined into the pGreen0179‐35S‐GWB‐YFP vector (Hellens et al., 2000). The constructed vectors were then transformed into Col‐0 to obtain transgenic materials using *Agrobacterium*‐mediated inflorescence transformation. For the construction of GUS genetic material, 2 kb promoter regions before ATG of *REK1* were cloned into the vector pGreen0179‐GUS (Hellens et al., 2000) for driving GUS reporter expression. The constructed vectors were then transformed into Col‐0 to obtain transgenic materials using *Agrobacterium*‐mediated inflorescence transformation.

For the complementation assays of *rbohd‐1*, the full‐length CDS of RBOHD and its CDS with serine 163 mutated to alanine were amplified using primer pairs RBOHD‐101‐F/RBOHD‐101‐R and RBOHDS163A‐101‐F/RBOHDS163A‐101‐R, and were constructed into the pEarleyGate101 vector using the self‐promoter‐driven cloning strategy. The constructed vectors were then transformed into *rbohd‐1* to obtain transgenic materials using *Agrobacterium*‐mediated inflorescence transformation. All primers used are listed in Data Set S1.

### Phylogenetic tree analysis

The complete protein sequences of REK1 and all members of its XI subfamily of LRR‐RLKs were aligned using ClustalW. The phylogenetic tree was reconstructed using MEGA11 with the maximum‐likelihood method and 1000 bootstrap replicates. The bootstrap value at a node indicates the branch's confidence level; higher values indicate greater confidence.

### Subcellular localization and GUS analysis

To investigate the subcellular localization, the 35S:REK1‐YFP and 35S:CBL1‐mCherry vectors were co‐transformed into Arabidopsis leaf protoplasts using polyethylene glycol (PEG)‐mediated protoplast transformation (D'Angelo et al., 2006; Yoo et al., 2007). After 12 h of cultivation at 21°C, images were acquired using a confocal microscope (TCS‐SP8; Leica, Wetzlar, Germany).

For spatial expression patterns, the ProREK1:GUS construct was introduced into Col‐0 via *Agrobacterium*‐mediated floral dip transformation, and stable transgenic lines were obtained after three generations of antibiotic selection. Seedlings and various tissues of Arabidopsis transgenic plants were incubated in GUS staining buffer at 37°C in darkness for 12 h. The stained tissues were then rinsed with 100% ethanol to remove the chlorophyll and imaged by a microscope (TS100; Nikon, Tokyo, Japan). The relative GUS staining intensity was analyzed with ImageJ, and the staining intensity in Mock‐Col‐0 cotyledons was set as ‘1’.

### RNA extraction and quantitative reverse transcription polymerase chain reaction

This experimental method is based on previous research (Liu et al., 2024). Total RNA was extracted using Total RNA Isolation Reagent (Biosharp, Hefei, China). cDNA was synthesized from 3 μg of total RNA using Hifair® IV Reverse Transcriptase (Yeasen Biotechnology, Shanghai, China) following the manufacturer's manual. The quantitative PCR assays were performed on the CFX Connect™ Real‐Time PCR Detection System (Bio‐Rad, Hercules, CA, USA) using 2 × SYBR Green qPCR Mix (Aidlab, Beijing, China). *EF‐1α* was used as a reference gene.

### Reactive oxygen species staining and quantification

NBT (Aladdin, CAS: 298‐83‐9, Shanghai, China) was dissolved in 50 mM sodium phosphate buffer (pH 7.0) to prepare a 0.2% solution. For NBT staining, 4‐day‐old seedlings were collected, transferred to 1/2 MS medium with or without 150 mM NaCl for 12 h, then incubated in NBT solution for 20 min. Finally, the solution is poured out, and the leaves are boiled in an ethanol solution for 10 min. The relative NBT staining intensity was analyzed with ImageJ, and the NBT staining intensity in Mock‐Col‐0 cotyledons was set as ‘1’. DAB (Aladdin, CAS: 91‐95‐2, Shanghai, China) was dissolved in water, and the pH value was adjusted to 3.7, resulting in a final concentration of 1 mg ml^−1^. The DAB staining protocol is identical to that used in the NBT staining experiment. The relative DAB staining intensity was analyzed with ImageJ, and the DAB staining intensity in Mock‐Col‐0 cotyledons was set as ‘1’.

The superoxide anion content was quantified using a superoxide anion (O^2−^) detection kit (Solarbio Corporation, BC1290, Beijing, China) in this study. Four‐day‐old seedlings were transferred to 1/2 MS medium containing or lacking 150 mM NaCl and treated for 12 h. Subsequently, 100 mg of seedlings was ground in liquid nitrogen, and superoxide anion content was measured according to the manufacturer's instructions. Hydrogen peroxide content was also quantitatively determined using a hydrogen peroxide detection kit (Solarbio Corporation, BC3590, Beijing, China). Sample processing was the same as above.

For H2DCF‐DA staining, 7‐day‐old seedlings were treated with 50 mM NaCl for 0 and 30 min, followed by 15 min of staining in the dark using a 20 μM H2DCF‐DA fluorescent probe, and then washed three times with ddH_2_O. The fluorescence was determined by the laser scanning confocal microscope (TCS SP8, Leica) with excitation at 488 nm and emission at 527 nm, and the relative fluorescence was analyzed with the software ImageJ, and the fluorescence in Mock‐Col‐0 was set as 1.

### Yeast two‐hybrid assay

The split‐ubiquitin membrane Y2H system was performed as described in previous studies (Stagljar et al., 1998). The pBT3‐STE‐REK1 construct was transformed into NMY51 yeast competent cells either alone or co‐transformed with pPR3‐N‐RBOHD. The transformed yeast cells were cultured in SD (‐Leu/Trp) liquid medium until the OD_600_ reached 0.8, then diluted with 0.9% sodium chloride solution and spotted onto SD double‐deficient (‐Leu/Trp) and SD quadruple‐deficient (‐Ade/His/Leu/Trp) medium plates.

### Bimolecular fluorescence complementation

The full‐length or truncated CDSs of REK1 and RBOHD were cloned into the vector pEarlyGate201‐N‐terminal YFP fragment (YN, nYFP) and pEarlyGate201‐C‐terminal YFP fragment (YC, cYFP) using the Gateway cloning strategy (Earley et al., 2006). YN and YC with different genes were transformed into *Agrobacterium tumefaciens* strain GV3101 and co‐infiltrated into *N. benthamiana* leaf epidermis at an OD_600_ of 0.6–0.8. After 3 days of infiltration, the YFP signal was detected with a 514 nm excitation laser and a 524–570 nm emission filter using a Leica SP8 inverted microscope. All primers used for BiFC experiments are shown in Data Set S1.

### Luciferase complementation imaging assay

LCI assay was performed as described (Chen et al., 2008). The coding regions of REK1 and RBOHD were cloned into the pJW771 and pJW772 vectors, respectively, at the *Bam*HI and *Sal*I restriction enzyme digestion sites. All constructs were transformed into *Agrobacterium* GV3101 and then injected into *N. benthamiana* leaves. After 48 h, 1 mM luciferin (LUCK‐1G; GOLDBIO, St. Louis, MO, USA) was uniformly applied to the leaf surface, and images were captured in the dark using a Lumazone imaging system, equipped with a 2048B CCD camera (Roper, Sarasota, FL, USA).

### Co‐IP assay

The CDSs of *REK1* and *RBOHD* were cloned into the FLAG‐tagged pUGW11 and pGreen0179‐35S‐GWB‐YFP vectors (Nakagawa et al., 2014), respectively, to generate REK1‐KD‐Flag and RBOHD‐YFP constructs. After 12 h of incubation, the constructs were transfected into Arabidopsis protoplasts using PEG‐mediated protoplast transformation (Yoo et al., 2007), and the total proteins were extracted using protein lysis solutions (50 mM Tris–HCl (pH 7.5), 150 mM NaCl, 10% glycerin, 10 mM DTT, 10 mM EDTA, 1 mM NaF, 1 mM Na_2_MoO_4_·2H_2_O, 1 mM PMSF (BL507A; Biosharp, Hefei, China), 1% SDS (w/v), 1% Triton X‐100 (v/v)). RBOHD‐YFP and YFP proteins were IP using anti‐GFP beads (1:300; Smart‐Lifesciences, Changzhou, China) at 4°C for 2 h. After washing with washing buffer (10 mM Na_2_HPO_4_, 1.8 mM KH_2_PO_4_, 390 mM NaCl, 2.7 mM KCl, and 0.2% Triton X‐100 (v/v) (pH 7.4)), the proteins bound to beads were subjected to Western blotting analysis using anti‐FLAG (1:3000; ABclonal, Wuhan, China) or anti‐GFP (1:3000; ABclonal, Wuhan, China) antibodies.

### Phosphorylation assay

*In vitro* phosphorylation assay, the construction of the recombinant protein vector is as follows: the CDS of RBOHD‐N, RBOHD‐N^S8,9A^, RBOHD‐N^S22,24,26A^, RBOHD‐N^S39A^, RBOHD‐N^S163A^, and RBOHD‐N^S343,347A^ was amplified and cloned into the pET‐28a vector. The CDS of REK1‐KD was cloned into the pGEX‐4T‐1 vector. His‐tagged RBOHD‐N and its variants, GST‐tagged REK1‐KD were expressed in *Escherichia coli* Rosetta (induced with 0.5 mM IPTG for 6 h at 25°C) and purified using standard procedure via Ni IDA Beads 6FF (Smart‐Lifesciences, SA052025, Changzhou, China) and Glutathione Agarose (Smart‐Lifesciences, SA010025). The recombinant proteins were incubated in kinase reaction buffer (20 mM HEPES, pH 7.4, 10 mM MgCl_2_, 1 mM DTT, and 20 mM ATP) for 30 min at 37°C, then stopped by adding 5× SDS loading buffer. The samples were then separated by 10% SDS‐PAGE, and the phosphorylation of RBOHD‐N was detected by immunoblot with a phosphoserine/threonine antibody (1:1000; ECM Biosciences, PP2551, Versailles, KY, USA).

For *in vivo* phosphorylation analysis, total protein from the YFP‐labeled plant was extracted using protein extraction buffer (50 mM Tris–HCl (pH 7.5), 150 mM NaCl, 10% glycerin, 10 mM DTT, 10 mM EDTA, 1 mM NaF, 1 mM Na_2_MoO_4_·2H_2_O, 1 mM PMSF (BL507A; Biosharp, Hefei, China), 1% SDS (w/v), 1% Triton X‐100 (v/v)). The total protein extracts were incubated with anti‐GFP magnetic beads (SMART, Changzhou, China) at 4°C for 4 h. The beads were washed three times with 50 mM Tris–HCl (pH 7.5), and the immunoprecipitated products were eluted with 5× SDS protein loading buffer and heated at 95°C for 10 min prior to Western blot analysis. Anti‐phosphoserine/threonine and anti‐GFP antibodies were used to detect the phosphorylated proteins and GFP‐tagged proteins, respectively.

### RNA‐seq analyses

For the RNA‐seq assay, total RNA was extracted from Col‐0 and *rek1‐1* seedlings grown normally on 1/2MS for 5 days, then transferred to 1/2 MS containing or lacking 150 mM NaCl for 12 h. The RNA‐seq analysis was performed by Novogene Science and Technology Co., Ltd. (Tianjin, China) using the Illumina Xplus Sequencing System (150 bp paired‐end reads; 6 G). The DEGs were identified using DESeq2 (|log2 fold change| ≥1, *P* < 0.05). Three independent biological replicates of each sample were used for RNA‐seq analysis.

### NADPH oxidase activity assay

For NADPH oxidase activity measurement, 4‐day‐old Arabidopsis seedlings of different genotypes were transferred to 1/2 MS medium with or without 150 mM NaCl and treated for 12 h. The seedlings were harvested and lysed in 1 ml of RIPA buffer (50 mM Tris–HCl, pH 7.4, 5 mM EDTA, 150 mM NaCl, 1% Triton X‐100, 0.1% SDS, 0.5% sodium deoxycholate, 1× protease inhibitor cocktail) and incubated for 1.5 h at 4°C. The lysed cells were then clarified by centrifugation at 13 000× ***g*** for 10 min, and the supernatants were used to detect NADPH oxidase activity as previously described (Ding et al., 2024).

### Cotyledon greening rate assay

Seeds of different genotypes were germinated for 7 days in 1/2 MS medium with or without 100 mM NaCl. Seedlings with green cotyledons were counted, and the cotyledon greening rate for each genotype was calculated as follows: (number of seedlings with green cotyledons/total number of seedlings for that genotype) × 100. The results for the cotyledon greening rate were plotted using GraphPad Prism software.

### MDA content assay

The MDA content was determined using the MDA content detection kit (Solarbio, BC6410, Beijing, China). Four‐day‐old Arabidopsis seedlings of different genotypes were transferred to 1/2 MS medium with or without 150 mM NaCl and treated for 12 h. Subsequently, 100 mg of seedlings was ground in liquid nitrogen, and MDA content was measured according to the manufacturer's instructions.

## AUTHOR CONTRIBUTIONS

SX and AL designed the experiments; AL performed the main experiments; RW performed the experiments, including Figure 3 and Figure S4; SW performed the experiments, including Figure 7F–I and Figure S7C; XL, SL, and HH assisted with experiments; SX supervised the research; AL wrote the manuscript with input from the authors.

## CONFLICT OF INTEREST

The authors declare that they have no competing interests.

## MATERIALS AVAILABILITY

The constructs produced in this study can be obtained by contacting the lead individual upon request.

## Supporting information


**Dataset S1.** List of differentially expressed genes identified in the RNA‐seq analysis.
**Figure S1.** Characterization of the *rek1* T‐DNA insertional mutant and complementation lines.
**Figure S2.** Overexpression of REK1 compensates for the salt stress‐sensitive phenotype of *rek1* mutant plants.
**Figure S3.** Characterization and phenotype of *rek2* and *rek3* mutants.
**Figure S4.** qRT‐PCR assay of Col‐0 and *rek1‐1* under salt stress.
**Figure S5.** The transcription of *REK1* is induced by the endogenous or exogenous application of H_2_O_2_.
**Figure S6.** REK1 does not interact with the C‐terminus of RBOHD.
**Figure S7.** Potential phosphorylation sites of RBOHD and identification of its point mutation materials.
**Figure S8.** Characterization of the *rek1 rbohd* T‐DNA insertional mutant.
**Figure S9.** The *rek1 rbohd* double mutation rescues excessive ROS accumulation in *rek1* under salt stress.
**Figure S10.** Germination rates of Col‐0, *rek1*, *rbohd*, and *rek1 rbohd* mutants under KCl and mannitol stresses.
**Figure S11.** REK1 kinase activity enhanced by salt treatment.
**Table S1.** Primers used in this study.

## Data Availability

The authors confirm that all data referred to here are available in the body of this article and its Supporting Information.
