# Supplementary material for: REK1 positively regulates salt stress by phosphorylating RBOHD to modulate ROS homeostasis in Arabidopsis
Source: Plant J. 2026 Aug 19;127(4):e71093. doi: 10.1111/tpj.71093 (PMC13489662; doi:10.1111/tpj.71093)
Supplement: Supplementary file 1 [file TPJ-127-0-s001.pdf]

Supplementary Material

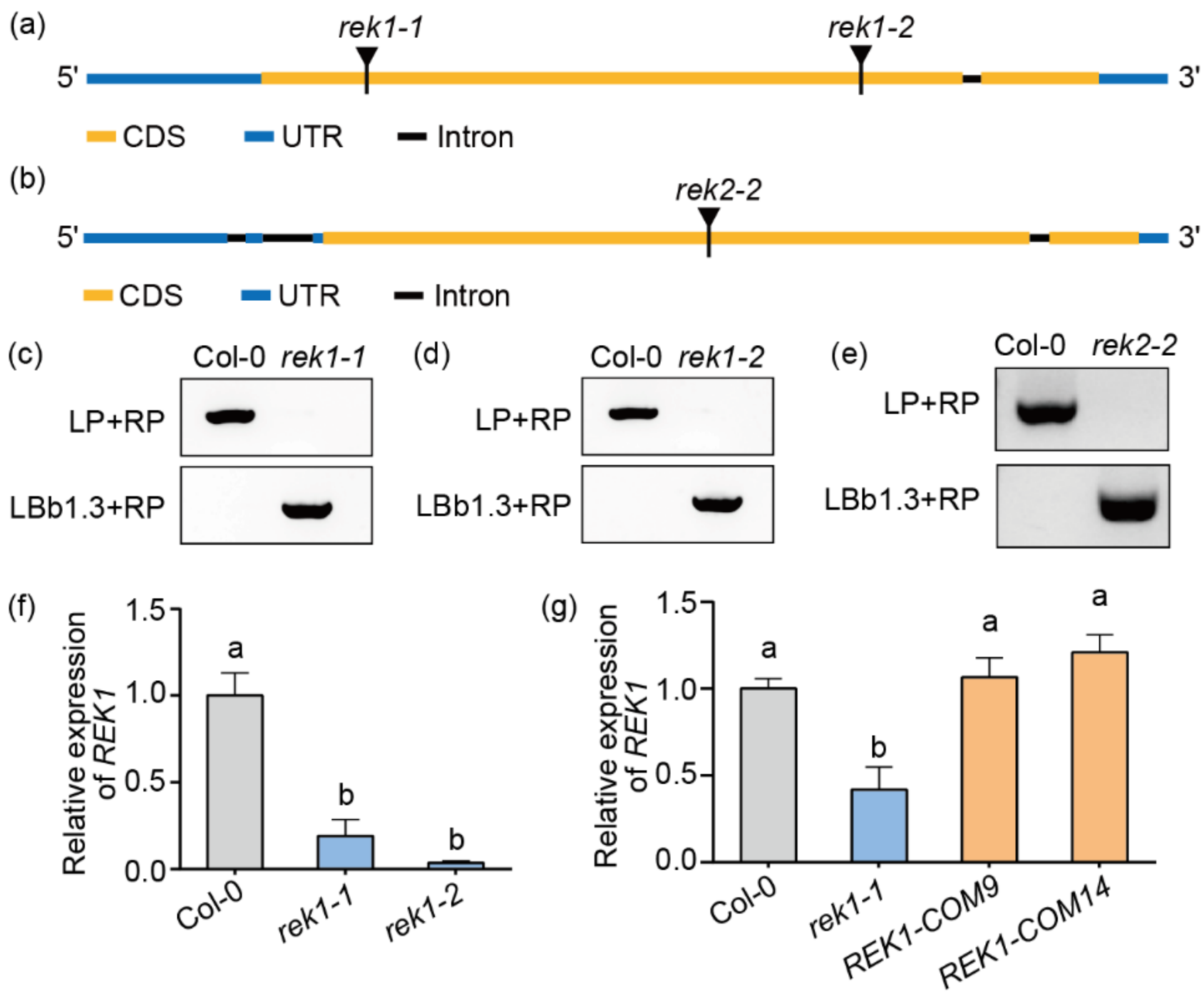

Figure S1. Characterization of the *rek1* T-DNA insertional mutant and complementation lines.

(a) T-DNA insertion sites of the *rek1-1* and *rek1-2* mutants are shown above the gene model. (b) T-DNA insertion site of the *rek2-2* mutant is shown above the gene model. (c-e) The mutants were identified by PCR using primers (*rek1-1*-LP/*rek1-1*-RP/LBb1.3, *rek1-2*-LP/*rek1-2*-RP/LBb1.3 and *rek2-2*-LP/*rek2-2*-RP/LBb1.3; Supplementary Data Set 1). (f) *REK1* transcripts in *rek1* were identified by qRT-PCR using primers (REK1-qF/REK1-qR; Supplementary Data Set 1). Significant differences were determined by one-way ANOVA with Tukey's test; different lowercase letters indicate significant differences ($P < 0.05$), n = 3. (g) Expression of *REK1* in indicated genotypes by qRT-PCR. Significant differences were determined by one-way ANOVA with Tukey's test; different lowercase letters indicate significant differences ($P < 0.05$), n = 3.

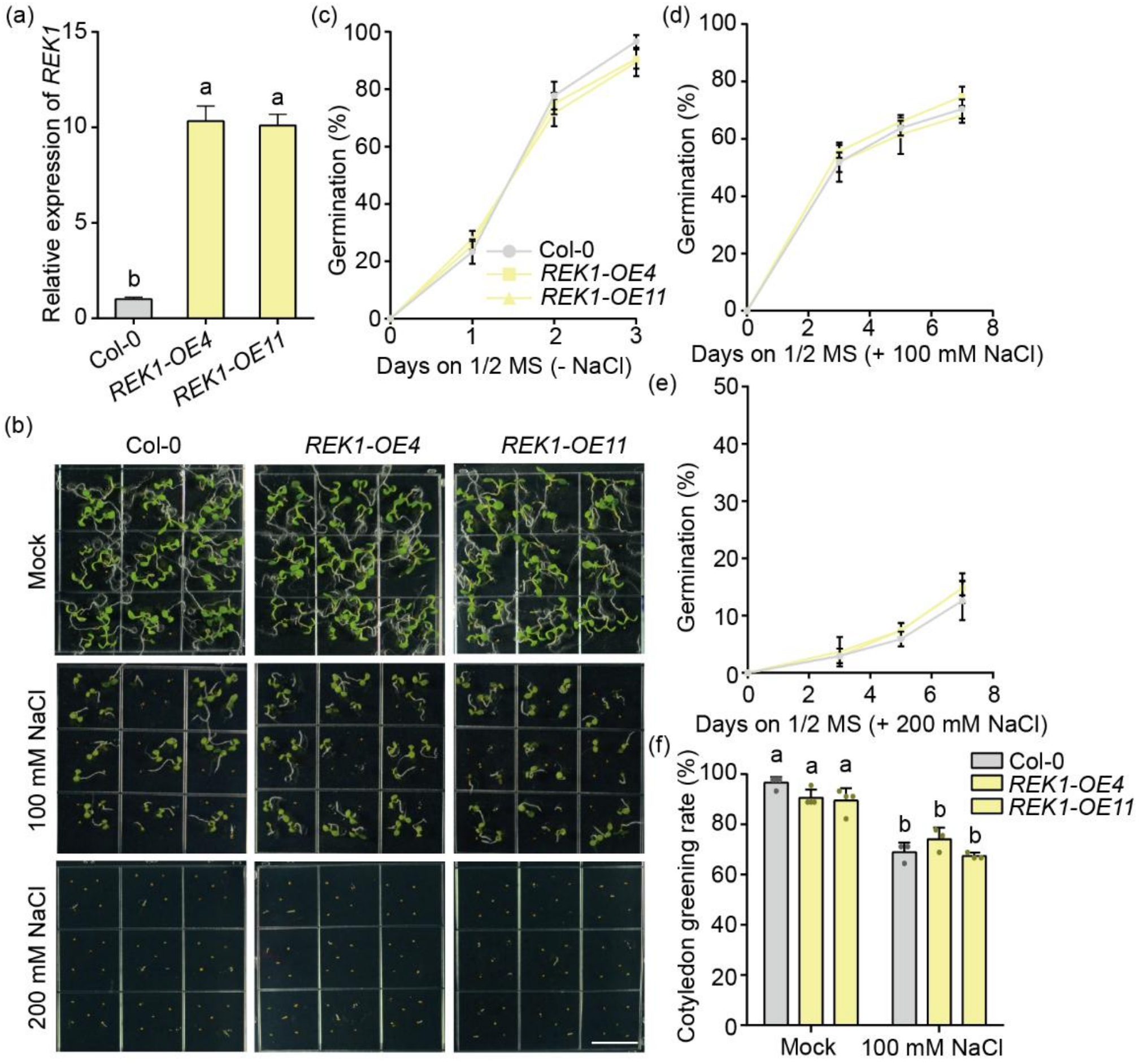

Figure S2. Overexpression of REK1 compensates for the salt stress-sensitive phenotype of *rek1* mutant plants.

(a) Expression of *REK1* in indicated genotypes by qRT-PCR. Significant differences were determined via one-way ANOVA with Tukey's test; different lowercase letters indicate significant differences ($P < 0.05$), n = 3. (b) Seed germination of Col-0, *REK1-OE4*, and *REK1-OE11* on 1/2 MS medium with or without NaCl treatment (100 and 200 mM). Seeds collected at the same time were analyzed. Bar, 1 cm. (c-e) The quantitative analysis of the germination rates is shown for control (c), 100 mM NaCl (d), and 200 mM NaCl (e) treatments. Percentages are the average of three repeats ± SD (n = 45). One-way ANOVA with Tukey's test is used for data significance analysis. (f) Statistics of cotyledon greening rate on the seventh day of the 100 mM NaCl treatment. Significant differences were determined via one-way ANOVA with Tukey's test; different lowercase letters indicate significant differences ($P < 0.05$).

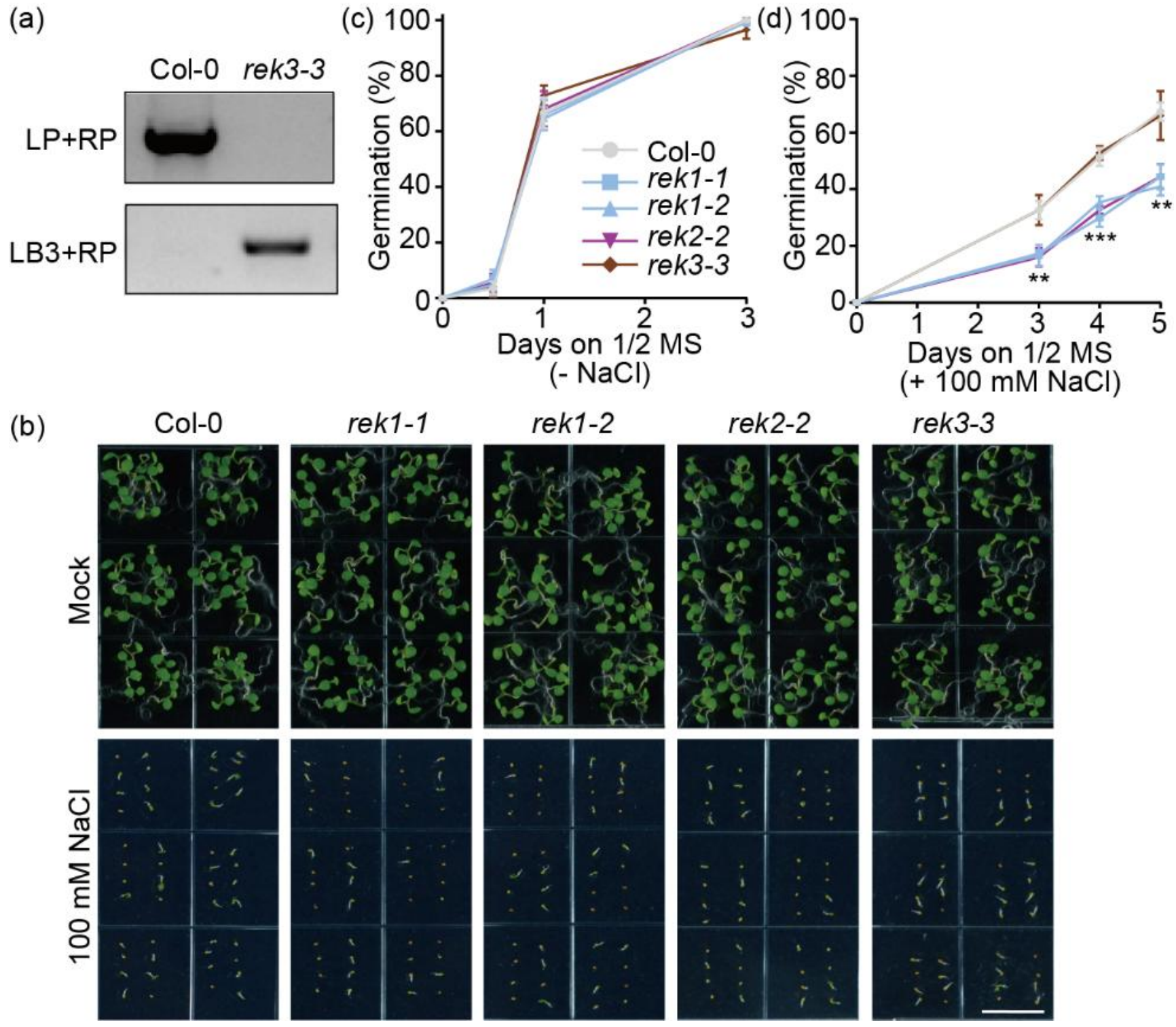

Figure S3. Characterization and Phenotype of *rek2* and *rek3* mutants.

(a) The *rek3-1* mutant was identified by PCR using primers (*rek3-3*-LP/ *rek3-3*-RP/LB3). (b) Seed germination of Col-0, *rek1-1*, *rek1-2*, *rek2-2*, and *rek3-3* on 1/2 MS medium with or without 100 mM NaCl treatment. Seeds collected at the same time were analyzed. Bar, 1 cm. (c-d) The quantitative analysis of the germination rates is shown for control (c) and 100 mM NaCl (d) treatment. Percentages are the average of three repeats ± SD (n = 48). Asterisks indicate a significant difference (**, $P < 0.01$; ***, $P < 0.001$) according to one-way ANOVA with Tukey's test.

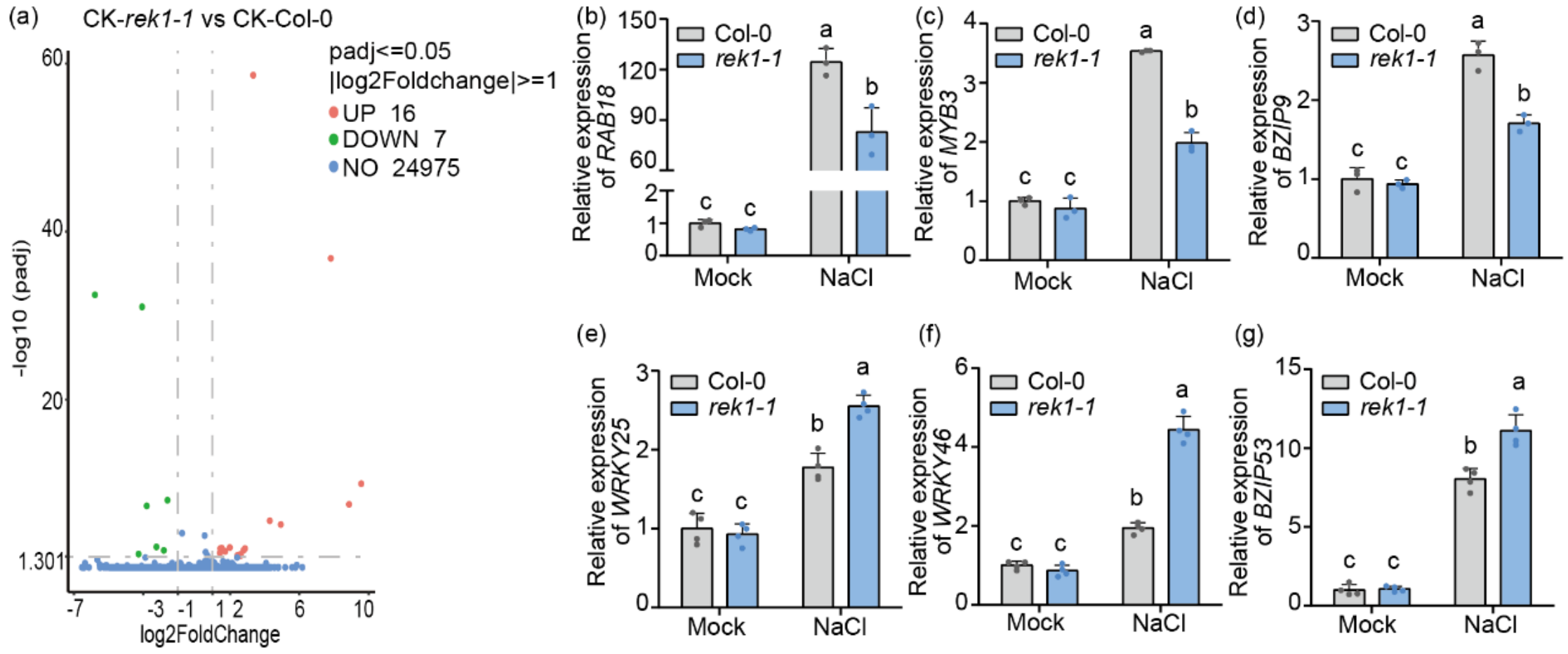

Figure S4. qRT-PCR assay of Col-0 and *rek1-1* under salt stress.

(a) Volcano plots showing DEGs (|log2 fold change| ≥ 1, $P < 0.05$) between *rek1-1* and Col-0 plants under normal growing conditions. The *REK1*-regulated genes were identified, comprising 7 down-regulated and 16 up-regulated genes in *rek1-1* relative to Col-0 plants. (b-g) Five-day-old Col-0 and *rek1-1* seedlings were transferred from 1/2 MS medium to the same medium supplemented with 150 mM NaCl. RNA was extracted 12 hours after treatment, and the expression levels of the *RAB18* (b), *MYB3* (c), *BZIP9* (d), *WRKY25* (e), *WRKY46* (f), and *BZIP53* (g) were detected by qRT-PCR. Significant differences were determined by two-way ANOVA with Tukey's test; different lowercase letters indicate significant differences ($P < 0.05$). Percentages are the average of three repeats ± SD ($n \geq 3$).

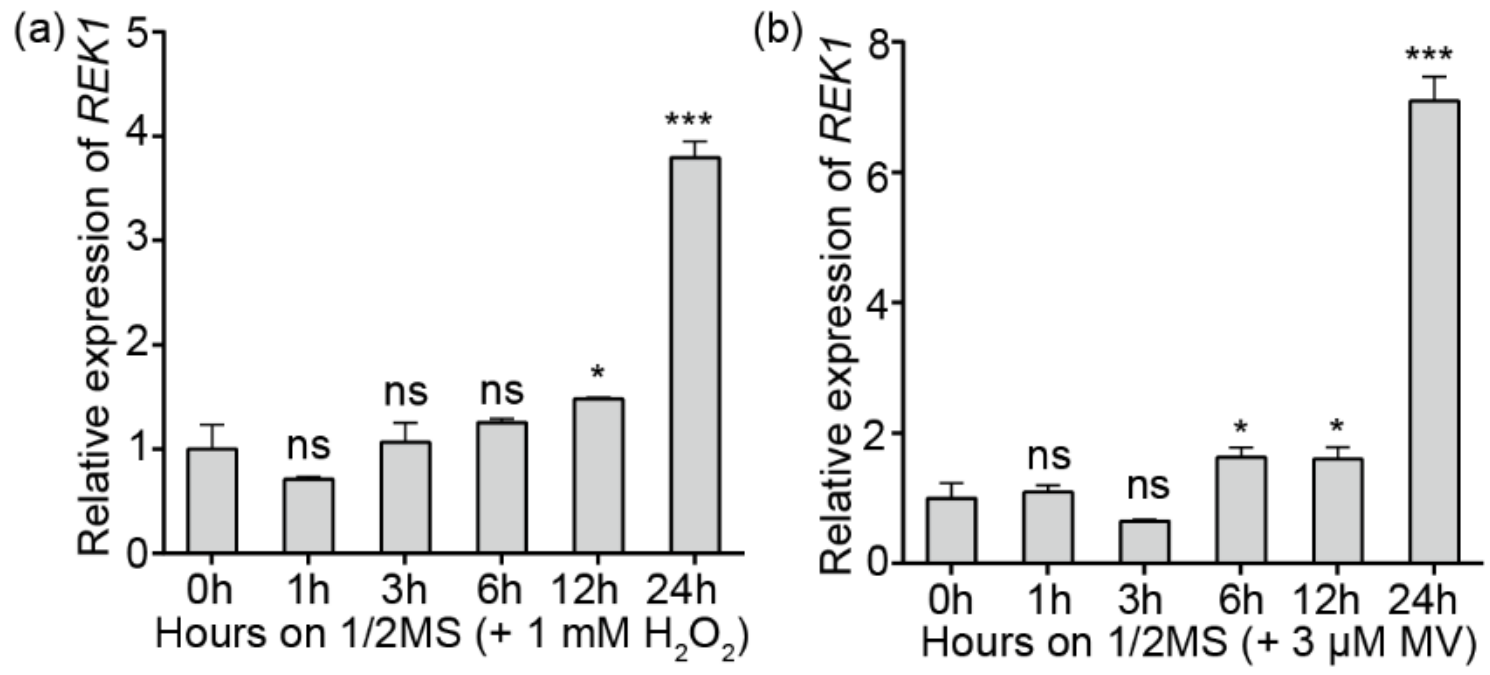

Figure S5. The transcription of *REK1* is induced by the endogenous or exogenous application of H_2O_2.

(a) The expression levels of *REK1* were analyzed by qRT-PCR after treating 5-day-old Col-0 seedlings with 1 mM H_2O_2 in 1/2 MS medium for 0, 1, 3, 6, 12, and 24 hours. (b) The expression levels of *REK1* were analyzed by qRT-PCR after treating 5-day-old Col-0 seedlings with 3 μM MV in 1/2 MS medium for 0, 1, 3, 6, 12, and 24 hours.

The mean ± SD data were obtained from three independent experiments for each treatment group (n = 3). Asterisks indicate a significant difference (ns, no significance; *, $P < 0.05$; ***, $P < 0.001$) according to one-way ANOVA with Tukey's test.

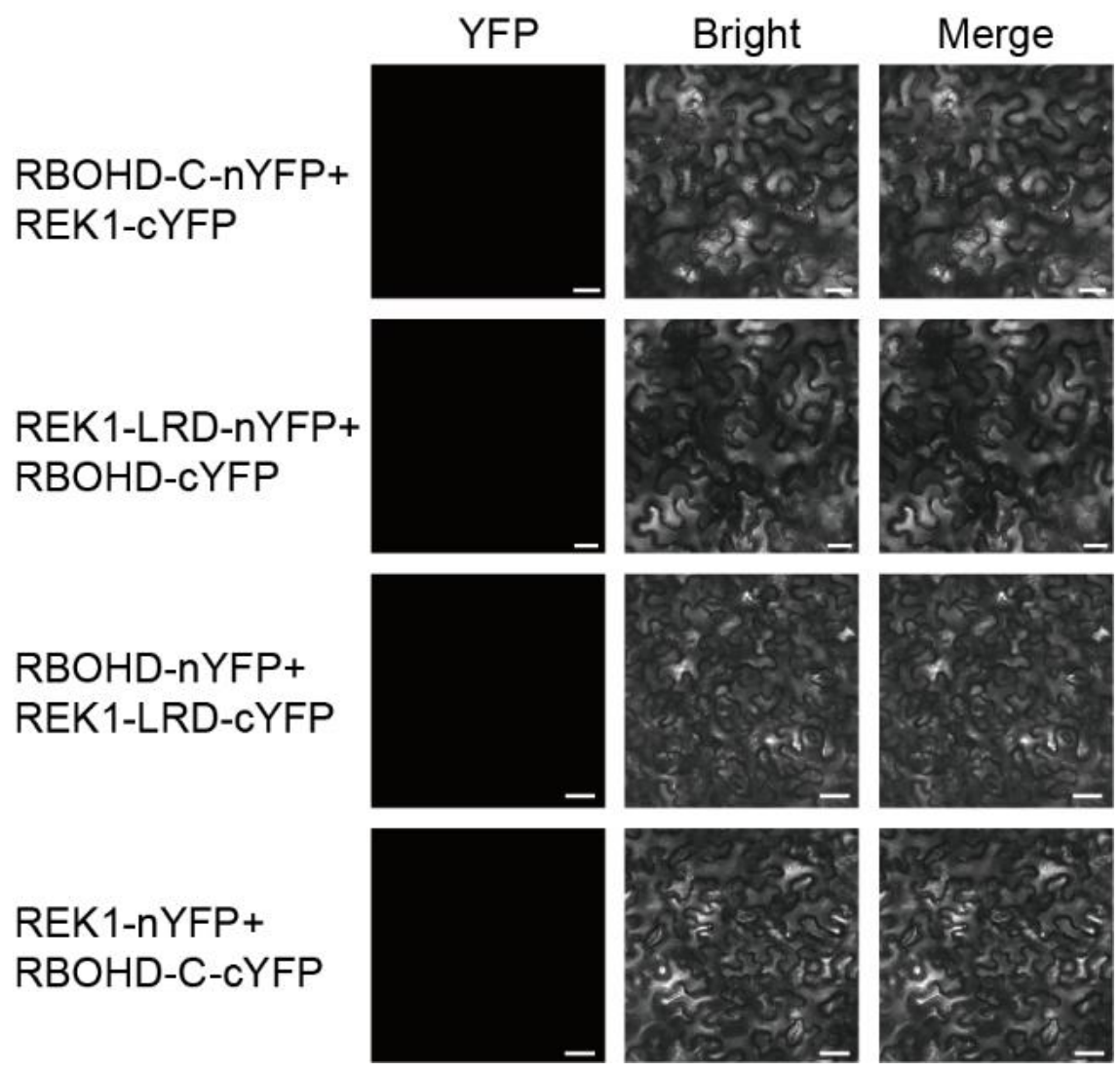

Figure S6. REK1 does not interact with the C-terminus of RBOHD.

BiFC assays show that REK1 does not interact with the C-terminus of RBOHD, which does not interact with REK1-LRD. Bars, 30 μm.

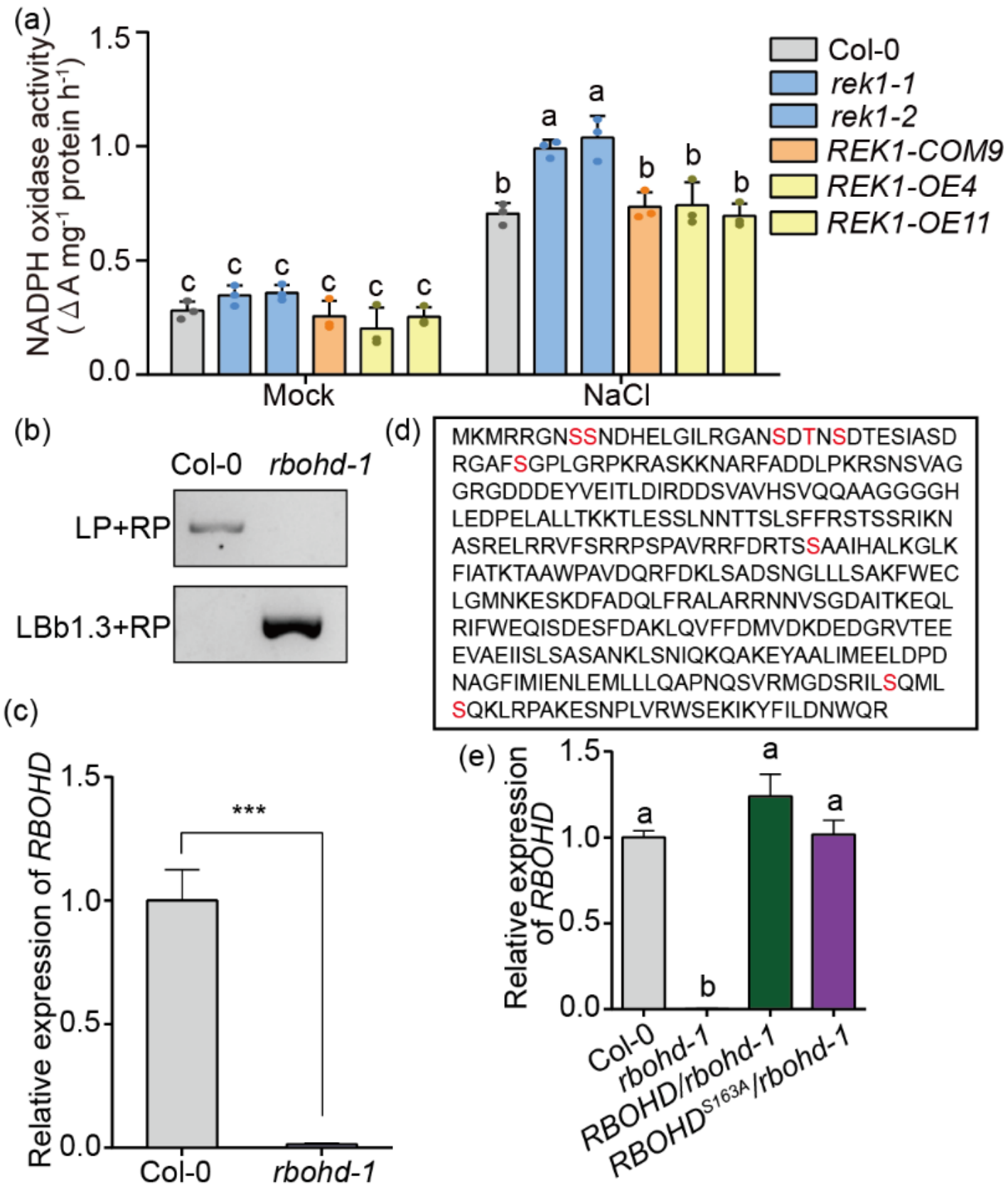

Figure S7. Potential phosphorylation sites of RBOHD and identification of its point mutation materials.

(a) NADPH oxidase activity in control and 150 mM NaCl-treated seedlings. Significant differences were determined via two-way ANOVA with Tukey's test; different lowercase letters indicate significant differences ($P < 0.05$). Mean ± SD values were obtained from three independent experiments (n = 3). (b-c) The *rbohd* mutant was identified by PCR (b) using primers (*rbohd-1*-LP/ *rbohd-1*-RP/LBb1.3), and *RBOHD* transcripts were identified by qRT-PCR (c) using primers (RBOHD-qF/RBOHD-qR). Asterisks indicate a significant difference (***, $P < 0.001$) according to Student's t-test. The average percentages of three repeats ± SD are shown (n = 3). (d) A schematic of the N-terminal of the RBOHD showing the location of the potential phosphorylation site identified by REK1. The red color indicates the predicted number of Ser/Thr residues. (e) Expression of *RBOHD* in indicated genotypes by qRT-PCR (RBOHD-qF/ RBOHD-qR). Significant differences were determined via one-way ANOVA with Tukey's test; different lowercase letters indicate significant differences ($P < 0.05$). The average percentages of three repeats ± SD are shown (n = 3).

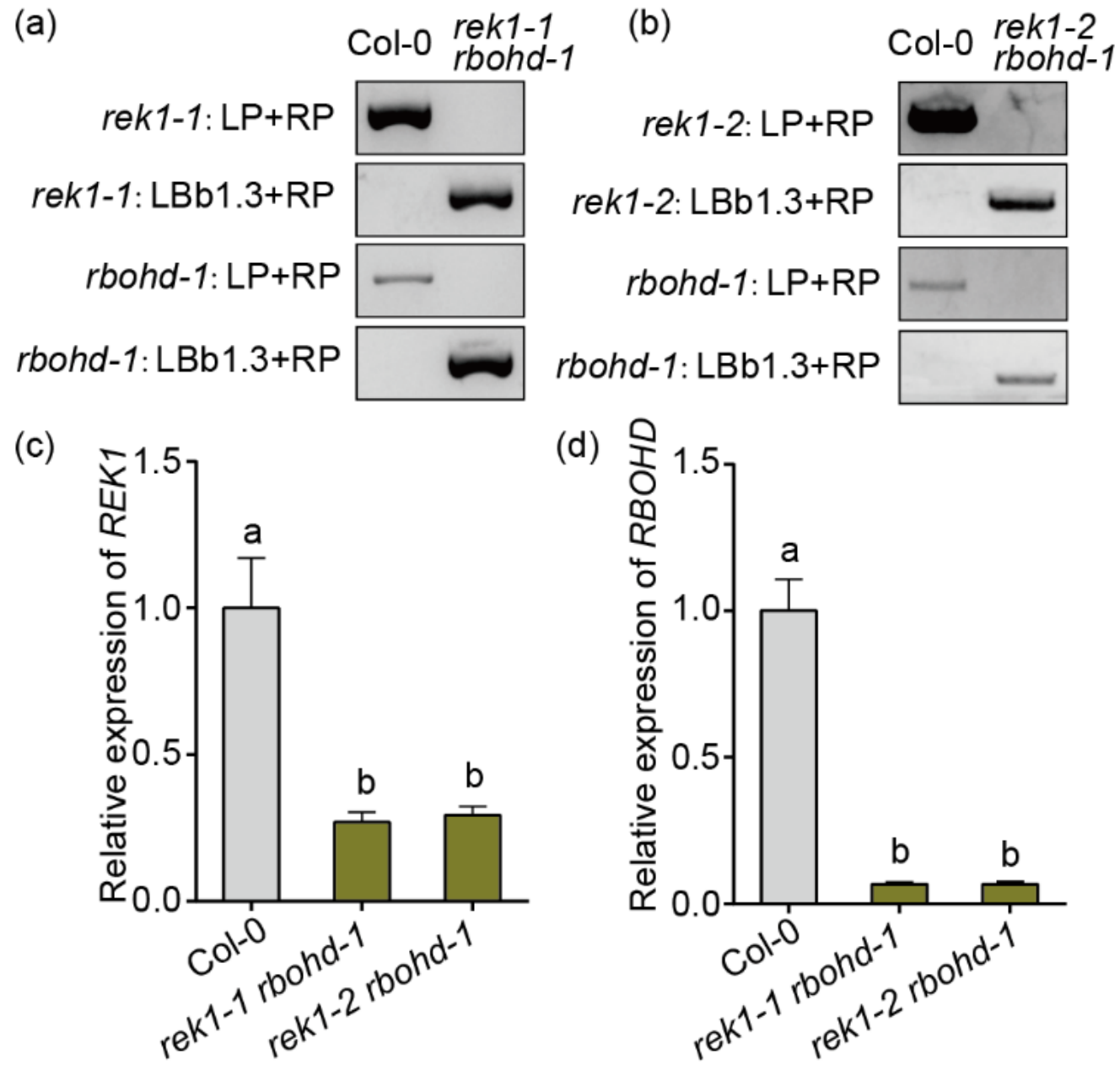

Figure S8. Characterization of the *rek1 rbohd* T-DNA insertional mutant.

(a-b) The *rek1 rbohd* double mutant was identified by PCR. (c-d) Expression of *REK1* (c) and *RBOHD* (d) in indicated genotypes by qRT-PCR. Significant differences were determined via one-way ANOVA with Tukey's test; different lowercase letters indicate significant differences ($P < 0.05$). The average percentages of three repeats ± SD are shown (n = 3).

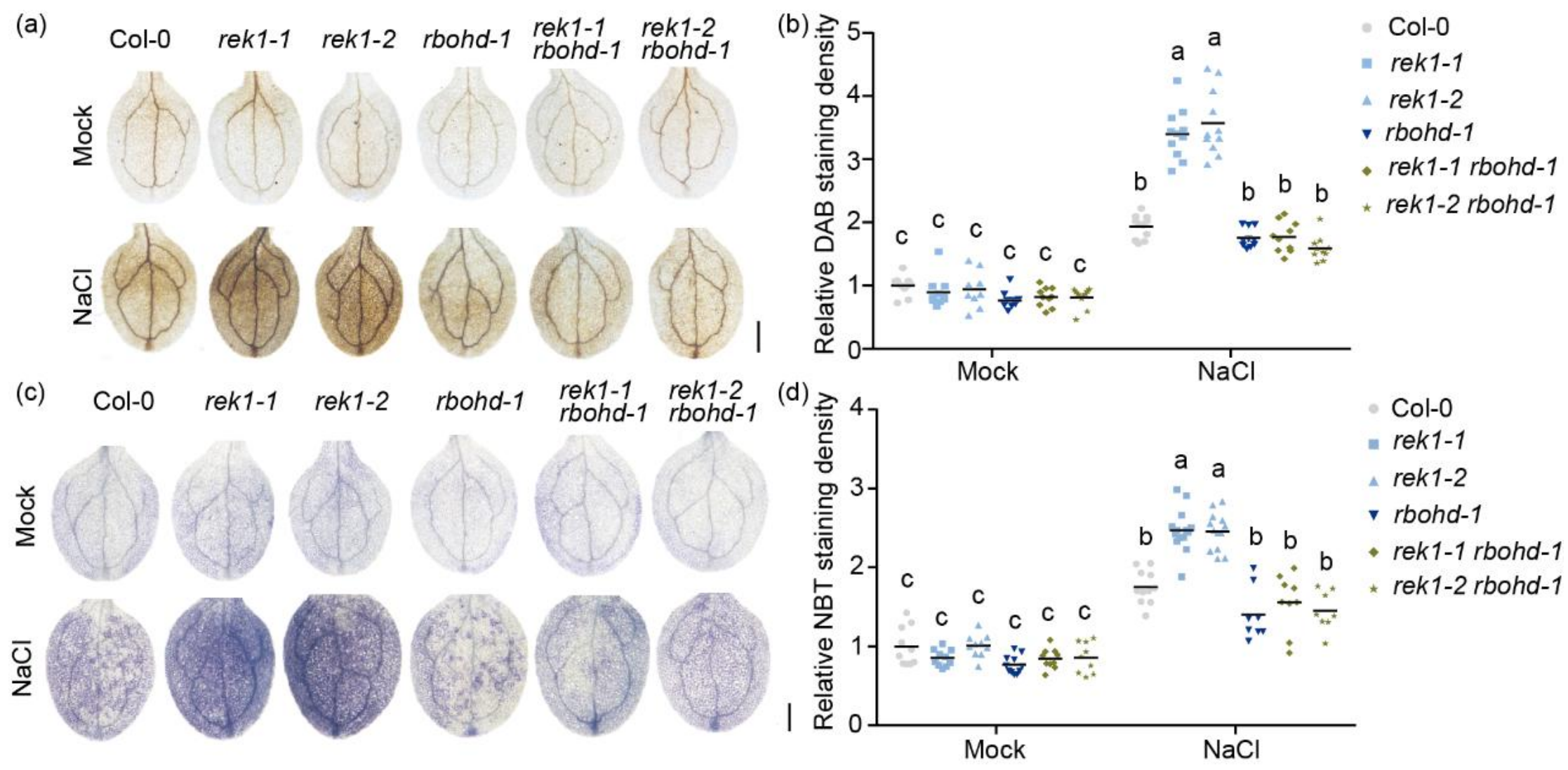

Figure S9. The *rek1 rbohd* double mutation rescues excessive ROS accumulation in *rek1* under salt stress.

(a-b) Four-day-old leaves were used for DAB staining (a) and staining was counted relatively quantitatively (b). Bars, 0.5 mm. Significant differences were determined via two-way ANOVA with Tukey's test; different lowercase letters indicate significant differences ($P < 0.05$). Mean ± SD values were obtained from three independent experiments for each treatment ($n \geq 9$). (c-d) Four-day-old leaves were selected for nitroblue tetrazolium chloride (NBT) staining (c) and staining was counted relatively quantitatively (d). Bars, 0.5 mm. Significant differences were determined via two-way ANOVA with Tukey's test; different lowercase letters indicate significant differences ($P < 0.05$). Mean ± SD values were obtained from three independent experiments for each treatment ($n \geq 8$).

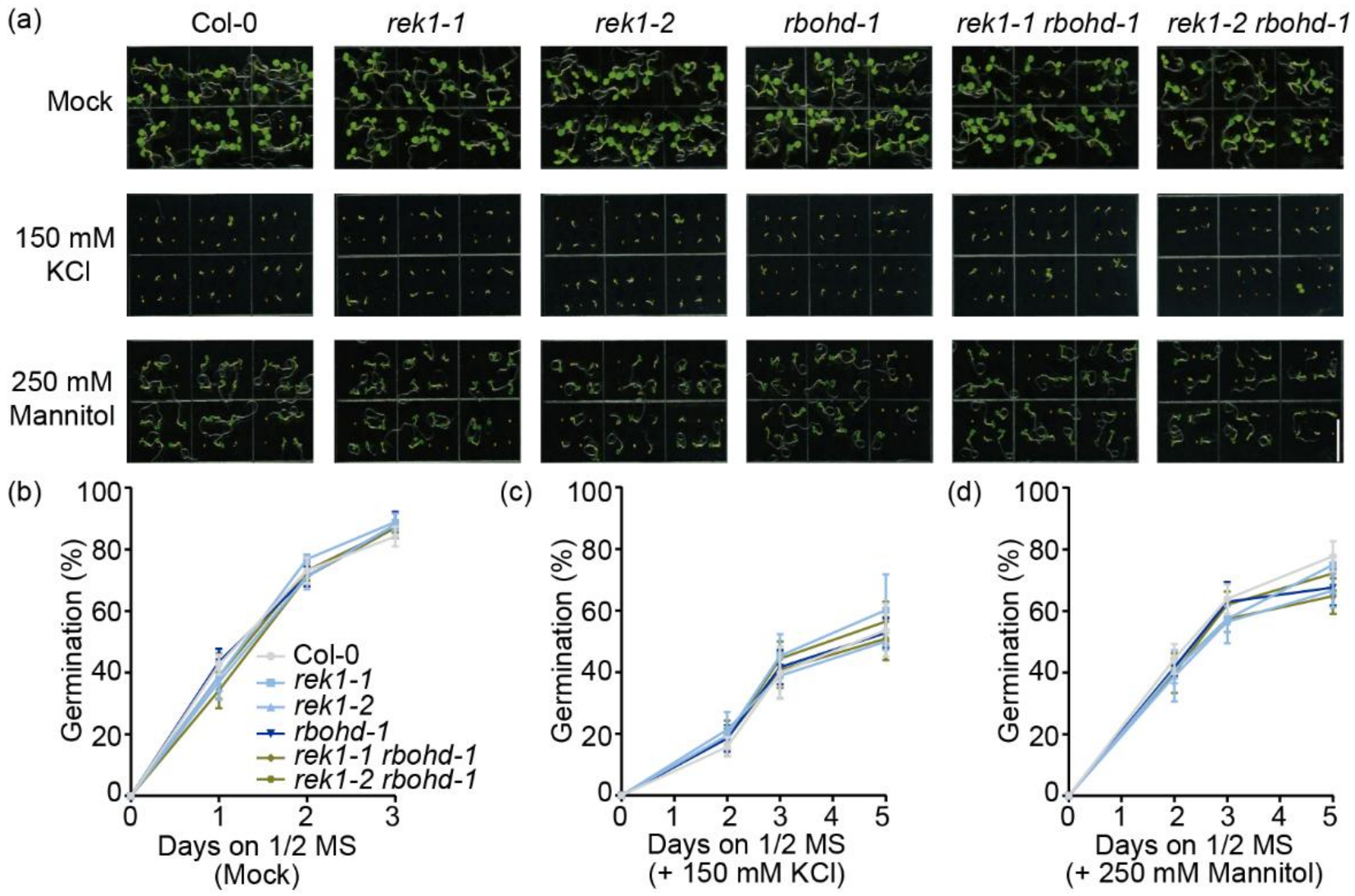

Figure S10. Germination rates of Col-0, *rek1*, *rbohd*, and *rek1 rbohd* mutants under KCl and Mannitol stresses.

(a) Seed germination of Col-0, *rek1*, *rbohd*, and *rek1 rbohd* on 1/2 MS medium supplemented with 150 mM KCl and 250 mM Mannitol, separately. The same batch of seeds was analyzed. Bar, 1 cm. (b-d) The quantitative analysis of the germination rates is shown for control (b), 150 mM KCl (c), and 250 mM Mannitol (d) treatments. Percentages are the average of three repeats ± SD (n = 36). One-way ANOVA with Tukey's test is used to analyze the significance of data.

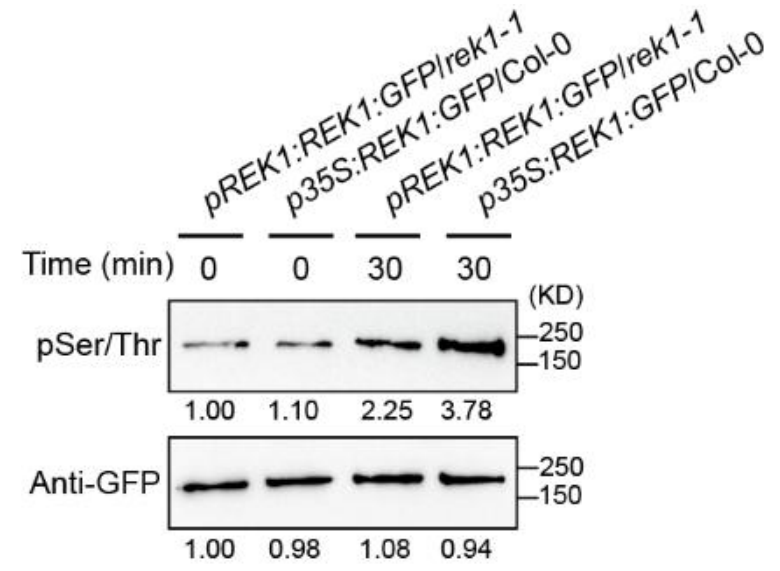

Figure S11. REK1 kinase activity is enhanced by salt treatment.

Seven-day-old *Pro35S:REK1:YFP/*Col-0 and *ProREK1:REK1:YFP/rek1-1* transgenic seedlings were subjected to 150 mM NaCl for 0 and 30 min. YFP-tagged REK1 proteins were enriched with anti-GFP beads. Anti-GFP and anti-pSer/Thr antibodies were used to detect total REK1 and phosphorylated REK1, respectively.

Table S1 Primers used in this study.

Primer name	Sequence (5'-3')
rek1-1-LP	GGTATCTTCCCTTCAAGCTGG
rek1-1-RP	TATCTCGCAAGCTGGAATCAG
rek1-2-LP	GCGACAAAGTCAAATTTCTGC
rek1-2-RP	AAACCAGATGTCATGGACAGC
rek2-1-LP	TAGGAAATGAACGACAATCGC
rek2-1-RP	TTTTCCACAATTCCCTCAGTG
rek3-1-LP	GTCTGGGGAAATACCAAGCTC
rek3-1-RP	AAAACTTTACCTGGGGCAATG
rbohd-1-LP	TTTGATGCCAAACTCCAAGTC
rbohd-1-RP	GCCGCCTAAGACTTTCTAAGC
LBb1.3	ATTTGCCGATTTCGGAAC
LB3	TAGCATCTGAATTTCATAACCAATCTCGATACAC
Ef-1α-F	TGAGCACGCTCTTCTTGCTTTCA
Ef-1α-R	GGTGGTGGCATCCATCTTGTTACA
RBOHD-qF	TAATGGACGAGATTTCGGAGTT
RBOHD-qR	CACCGTTCTTAGCGTGTTGC
REK1-qF	TGAAAACTACTTCACGGGATCA
REK1-qR	CTGGTTTGTGTAAAGGTACAGC
RAB18-qF	GGAGGAAGAAGGGAATAACACA
RAB18-qR	GGGAAGCTTTTCCTTGATCTTG
MYB3-qF	GGAAAGTGAGTGTTGTTGACTC
MYB3-qR	CAATTCCGACACGACTCATTAC
BZIP9-qF	GAGTATCTCACTACAAGCCCTC
BZIP9-qR	ACTATCAGCTGACAGATTCTCG
WRKY25-qF	GTTCAAGATCACGAGAAGAAGC
WRKY25-qR	GTTTTCGCTCTTCTTCACTTGT
WRKY46-qF	GTTTAGCAGTGAAGCAAGTTCA
WRKY46-qR	CTTCCGATTGCTCTGTAACATG
BZIP53-qF	AATGCAAACAAGTCCTGAATCC
BZIP53-qR	TCAAATCACCAAGTTGCTTCTG
REK1-GUS-F	GGGGACAAGTTTGTACAAAAAAGCAGGCTTCTTAAAAGATTGTGATTTGTGAGCAT
REK1-GUS-R	GGGGACCACTTTGTACAAGAAAGCTGGGTCACAAGTTAGCTTTGGTAAGAACACA

REK1-OE(GW)-F	GGGGACAAGTTTGTACAAAAAAGCAGGCTTCAAAAGATTGTGATTTGTGAGCATTT
REK1-OE(GW)-R	GGGGACCACTTTGTACAAGAAAGCTGGGTCTCAAATTTCTGCAGAATAGCAAGAA
REK1-COM-F	GGGGACAAGTTTGTACAAAAAAGCAGGCTTCATGAGAGGGAGGATATGTTTCTTGG
REK1-COM-R	GGGGACCACTTTGTACAAGAAAGCTGGGTCAATTTCTTTGGAGGAGTTTGCTTCT
RBOHD-101-F	TTTGAAAAATCTCAGAATTCAACATCTGTTCATGATGCCTGCAT
RBOHD-101-R	GACCATGGAGGCCTGGATCCGAAGTTCTCTTTGTGGAAGTCAAA
RBOHDS163A-101-F	CGGTTTGACCGCACGAGCGCCGCG
RBOHDS163A-101-R	GAGTGCGTGGATGGCCGCGGCGCT
RBOHD-OE-YFP-F	ACACGATCGATAAGCTTCCCATGAAAATGAGACGAGGCAAT
RBOHD-OE-YFP-R	TCGCCCTTGCTCACCATCCCGAAGTTCTCTTTGTGGAAGTCAAA
REK1-KD-GST-F	CGCGTGGATCCCCGGAATTCATGGATGTCATGGACAGCTACTAT
REK1-KD-GST-R	TCACGATGCGGCCGCTCGAGAAGGCTACTTGATCCCCGAGCT
RBOHD-N-His-F	CACAGAGAACAGATTGGTGGATCCATGAAAATGAGACGAGGCAAT
RBOHD-N-His-R	GTGGTGGTGGTGGTGGTGCTCGAGTCATCTCTGCCAATTGTCAAGTATGAA
RBOHD-$N^{S8,9A}$-His-F	ATGAAAATGAGACGAGGCAATGCAGCT
RBOHD-$N^{S8,9A}$-His-R	TTCATGGTCGTTAGCTGCATT
RBOHD-$N^{S22,24,26A}$-His-F	GGAGCTAACGCGGACGCCAACGCG
RBOHD-$N^{S22,24,26A}$-His-R	CGTGTCCGCGTTGGCGTCCGCGTT
RBOHD-N^{S39A}-His-F	GACCGTGGTGCCTTTGCC
RBOHD-N^{S39A}-His-R	CCGGCCAAGCGGACCGGCAAA
RBOHD-N^{S163A}-His-F	CGGTTTGACCGCACGAGCGCCGCG
RBOHD-N^{S163A}-His-R	GAGTGCGTGGATGGCCGCGGCGCT
RBOHD-$N^{S343,347A}$-His-F	AGGATACTTGCTCAGATGTTAGCT
RBOHD-$N^{S343,347A}$-His-R	AAGCTTCTGAGCTAACATCTGAGC

REK1-KD(GW)-F	GGGGACAAGTTTGTACAAAAAAGCAGGCTTCATGGATGTCATGGACAGCTACTAT
REK1-KD(GW)-R	GGGGACCACTTTGTACAAGAAAGCTGGGTCAAGGCTACTTGATCCCCGAGCT
REK1-LRD(GW)-F	GGGGACAAGTTTGTACAAAAAAGCAGGCTTCATGAACGAAGAAGGGCGTGTTC
REK1-LRD(GW)-R	GGGGACCACTTTGTACAAGAAAGCTGGGTCCCGCTGGAACACGGCTGTGTCT
RBOHD-N(GW)-F	GGGGACAAGTTTGTACAAAAAAGCAGGCTTCATGAAAATGAGACGAGGCAATTC
RBOHD-N(GW)-R	GGGGACCACTTTGTACAAGAAAGCTGGGTCTCTCTGCCAATTGTCAAGTATGAA
RBOHD-C(GW)-F	GGGGACAAGTTTGTACAAAAAAGCAGGCTTCATGCTCCGTGCTTTCAGATCAA
RBOHD-C(GW)-R	GGGGACCACTTTGTACAAGAAAGCTGGGTCGAAGTTCTCTTTGTGGAAGTCAAAC
REK1-JW771-F	ACGGGGGACGAGCTCGGTACATGAGAGGGAGGATATGTTTCTTG
REK1-JW771-R	GGGACGCGTACGAGATCTGGTCAATTTCTTTGGAGGAGTTTGCTT
RBOHD-JW772-F	TACGCGTCCCGGGGCGGTACCATGAAAATGAGACGAGGCAAT
RBOHD-JW772-R	ACGAACGAAAGCTCTGCAGGCTAGAAGTTCTCTTTGTGGAAGTCAAA
REK1-KD-pBT3-STE-F	CTGCAATGGCCATTACGGCCATGCGGCGGGAACCAGCGTTTGT
REK1-KD-pBT3-STE-R	TCCTGCAGATGGCCGAGGCGGCCAATTTCTTTGGAGGAGTTTGCT
RBOHD-pPR3-N-F	CCAGATTACGCTGGATCCATGAAAATGAGACGAGGCAAT
RBOHD-pPR3-N-R	AAGCTTGATATCGAATTCGAAGTTCTCTTTGTGGAAGTCAAA

Dataset S1

gene_id	CK_WT_1	CK_WT_2	CK_WT_3	CK_rek1_1	CK_rek1_2	CK_rek1_3	NaCl_WT_1	NaCl_WT_2	NaCl_WT_3	NaCl_rek1_1	NaCl_rek1_2	NaCl_rek1_3	gene_name	gene_chr	gene_start	gene_end	gene_strand	gene_length	gene_biotype	gene_description	Family
AT1G02850	1.521273	1.818331	1.835239	4.349499	4.564936	2.223997	79.59787	145.875	98.57285	81.34286	57.63509	69.5058	BGLU11	1	630374	633420	+	2196	protein_coding	beta glucosidase 11 [Source:NCBI gene (formerly Entrezgene);Acc:839435] && sp|B3H5Q1|BGL11_ARATH Beta-glucosidase 11 OS=Arabidopsis thaliana OX=3702 GN=BGLU11 PE=2 SV=2 && PF00232:Glycosyl hydrolase family 1	B3
AT2G37760	7.124895	5.416985	6.981833	8.244455	8.805794	8.2228	61.84522	74.93155	59.05439	48.6009	44.31839	45.11214	AKR4C8	2	15831656	15833921	+	1665	protein_coding	NAD(P)-linked oxidoreductase superfamily protein [Source:NCBI gene (formerly Entrezgene);Acc:818353] && sp|O80944|AKRC8_ARATH Aldo-keto reductase family 4 member C8	FAR1

																				OS=Arabidopsis thaliana OX=3702 GN=AKR4C8 PE=1 SV=2 && PF00248:Aldo/keto reductase family	
AT2G42540	0.832137	2.362212	0.971696	4.62958	7.83468	1.501696	213.9832	143.5635	105.9831	21.02359	30.82156	69.34301	COR15A	2	17710441	17712330	-	1584	protein_coding	cold-regulated 15a [Source:NCBI gene (formerly Entrezgene);Acc:818854] && sp|Q42512|CR15A_ARATH Protein COLD-REGULATED 15A, chloroplastic OS=Arabidopsis thaliana OX=3702 GN=COR15A PE=1 SV=1 && -	-
AT1G52030	0.5831	0.422526	0.326433	2.13755	0.634825	0.499444	9.512302	7.204008	5.664293	4.905404	4.597594	4.329239	MBP2	1	19345871	19349088	-	3040	protein_coding	myrosinase-binding protein 2 [Source:NCBI gene (formerly Entrezgene);Acc:841632] && sp|Q9SAV1|JAL6_ARATH Myrosinase-binding protein 2 OS=Arabidopsis thaliana OX=3702 GN=F-ATMBP	B3

																			PE=2 SV=1 && PF01419:Jacalin-like lectin domain	
AT2G44460	0.395053	0.587594	0.45396	0.107444	0.122615	0.266248	5.112837	9.068459	6.461547	12.89025	11.05582	11.53592	BGLU28	2	18346306	18350026	+	2186	protein_coding	beta glucosidase 28 [Source:NCBI gene (formerly Entrezgene);Acc:819053] && sp|Q4V3B3|BGL28_ARATH Beta-glucosidase 28 OS=Arabidopsis thaliana OX=3702 GN=BGLU28 PE=2 SV=1 && PF00232:Glycosyl hydrolase family 1	EIL
AT5G24770	4.047499	1.068312	2.217587	4.492952	6.877741	6.159372	187.6385	89.04936	86.2092	48.02177	44.81677	68.61814	VSP2	5	8500468	8502224	-	1516	protein_coding	vegetative storage protein 2 [Source:NCBI gene (formerly Entrezgene);Acc:832546] && sp|O82122|VSP2_ARATH Vegetative storage protein 2 OS=Arabidopsis thaliana OX=3702 GN=VSP2 PE=2 SV=1 && PF03767:HAD	MYB

																				superfamily, subfamily IIIB (Acid phosphatase)	
AT1G76600	2.169703	1.676507	1.734193	4.032704	3.8781	2.889173	13.56997	25.76179	23.85041	141.4181	58.60877	72.26528	AT1G76600	1	28746833	28747848	+	1016	protein_coding	poly polymerase [Source:NCBI gene (formerly Entrezgene);Acc:843993] && - && PF14009:Domain of unknown function (DUF4228)	-
AT4G23600	2.233244	6.116569	1.613161	10.46139	13.62233	3.571206	106.7626	99.92782	63.81536	54.56563	61.62911	67.89022	CORI3	4	12310379	12313212	+	2310	protein_coding	Tyrosine transaminase family protein [Source:NCBI gene (formerly Entrezgene);Acc:828460] && sp|Q9SUR6|CORI3_ARATH Cystine lyase CORI3 OS=Arabidopsis thaliana OX=3702 GN=CORI3 PE=1 SV=1 && PF00155:Aminotransferase class I and II	M-type_MADS
AT1G54020	0.115287	0.10624	0.11558	0.430261	0.5099	0.288835	3.921315	2.082321	3.433984	6.352478	4.477577	5.587498	AT1G54020	1	20161555	20163750	-	1577	protein_coding	GDSL-like Lipase/Acylhydrolase superfamily protein [Source:NCBI gene	ERF

																			(formerly Entrezgene);Acc:841840] && sp|Q9C5N8|GDL20_ARATH GDSL esterase/lipase At1g54020 OS=Arabidopsis thaliana OX=3702 GN=At1g54020 PE=2 SV=1 && PF00657:GDSL-like Lipase/Acylhydrolase	
AT1G49500	60.4275	59.64046	63.40861	86.84377	84.55942	69.16392	18.12054	19.41128	23.2256	12.87741	13.54291	15.52885	AT1G49500	1	18320490	18322505	-	1428	protein_coding	transcription initiation factor TFIID subunit 1b-like protein [Source:NCBI gene (formerly Entrezgene);Acc:841374] && - && -	-
AT2G40330	10.59225	17.046	15.09777	7.470203	10.24113	5.212203	1.466698	1.217053	2.28282	5.579187	3.457812	2.20169	PYL6	2	16844588	16846194	-	1607	protein_coding	PYR1-like 6 [Source:NCBI gene (formerly Entrezgene);Acc:818626] && sp|Q8S8E3|PYL6_ARATH Abscisic acid receptor PYL6 OS=Arabidopsis thaliana OX=3702 GN=PYL6 PE=1 SV=1 &&	MYB_related

AT3G52310	0.204655	0.41148	0.547132	1.121647	1.239797	0.683644	3.682803	6.888899	7.50817	5.289635	3.198263	2.896533	AT3G52310	3	19399188	19402981	+	2443	nontranslating_CDS	PF10604:Polyketide cyclase / dehydrase and lipid transport - && sp|Q9FT51|AB27G_ARATH ABC transporter G family member 27 OS=Arabidopsis thaliana OX=3702 GN=ABCG27 PE=2 SV=1 && -	G2-like
AT1G47960	3.295075	3.785971	3.530402	5.444696	4.316485	4.635313	13.31011	9.995026	12.44334	6.05021	9.050728	9.939682	C/VIF1	1	17681157	17683719	-	1807	protein_coding	cell wall / vacuolar inhibitor of fructosidase 1 [Source:NCBI gene (formerly Entrezgene);Acc:841214] && sp|F4HWQ8|CVIF1_ARATH Cell wall / vacuolar inhibitor of fructosidase 1 OS=Arabidopsis thaliana OX=3702 GN=C/VIF1 PE=1 SV=1 && PF04043:Plant invertase/pectin methylesterase inhibitor	LSD

AT1G52890	0.033396	0.041034	0.119043	0.402672	0.413577	0.148745	2.029747	4.648188	3.538909	6.318739	6.650166	6.507389	NAC019	1	19696923	19698482	-	1361	protein_coding	NAC domain containing protein 19 [Source:NCBI gene (formerly Entrezgene);Acc:841722] && sp|Q9C932|NAC19_ARATH NAC domain-containing protein 19 OS=Arabidopsis thaliana OX=3702 GN=NAC019 PE=1 SV=1 && PF02365:No apical meristem (NAM) protein	NAC
AT1G13990	8.653207	6.969768	8.564515	13.87829	11.69734	13.9802	27.86218	39.55832	28.94309	23.81724	20.56167	22.30288	AT1G13990	1	4794664	4796599	+	1258	protein_coding	plant/protein [Source:NCBI gene (formerly Entrezgene);Acc:837959] && - && -	MYB
AT3G01170	9.008043	9.0650 1	9.289587	10.38206	10.62438	11.36899	21.10156	19.859	21.93572	16.87211	16.21189	18.27357	AT3G01170	3	57545	58924	+	1380	protein_coding	Ribosomal protein L34e superfamily protein [Source:NCBI gene (formerly Entrezgene);Acc:820469] && sp|P0C8Q9|Y5902_ARATH Uncharacterized protein At5g19025 OS=Arabidopsis thaliana	-

																				OX=3702 GN=At5g19025 PE=2 SV=3 && -	
AT2G29350	0	0	0	0.038153	0.431054	0.055494	2.871568	3.265323	3.028254	9.563363	10.28783	8.846277	SAG13	2	12600914	12602556	+	1368	protein_coding	senescence-associated 13 [Source:NCBI gene (formerly Entrezgene);Acc:817484] && sp|Q9ZW18|SAG13_ARATH Senescence-associated protein 13 OS=Arabidopsis thaliana OX=3702 GN=SAG13 PE=1 SV=1 && PF00106:short chain dehydrogenase	C2H2
AT1G21410	3.579444	4.242849	3.314903	6.060821	5.165391	4.829708	9.940321	11.02143	9.911385	5.679105	8.258221	8.644046	SKP2A	1	7496998	7499616	+	1619	protein_coding	F-box/RNI-like superfamily protein [Source:NCBI gene (formerly Entrezgene);Acc:838740] && sp|Q9LPL4|SKP2A_ARATH F-box protein SKP2A OS=Arabidopsis thaliana OX=3702 GN=SKP2A PE=1 SV=1 && PF00646:F-box domain	-

AT1G74710	3.440168	3.176615	3.223627	3.663156	4.192695	4.248482	8.986624	9.348167	12.82129	24.4036	10.96381	11.90881	EDS16	1	28070295	28074177	+	2180	protein_coding	ADC synthase superfamily protein [Source:NCBI gene (formerly Entrezgene);Acc:843810] && sp|Q9S7H8|ICS1_ARATH Isochorismate synthase 1, chloroplastic OS=Arabidopsis thaliana OX=3702 GN=ICS1 PE=1 SV=2 && PF00425:chorismate binding enzyme	-
AT1G58370	2.800685	2.148617	2.725539	3.95215	3.894842	3.196116	0.380702	0.458932	0.688539	1.500481	0.967033	1.050103	RXF12	1	21684263	21688359	+	3262	protein_coding	glycosyl hydrolase family 10 protein / carbohydrate-binding domain-containing protein [Source:NCBI gene (formerly Entrezgene);Acc:842206] && sp|A0A1P8AWH8|XYN1_ARATH Endo-1,4-beta-xylanase 1 OS=Arabidopsis thaliana OX=3702 GN=XYN1 PE=1 SV=1 && PF02018:Carbohydrate	-

																			binding domain|PF00331:Glycosyl hydrolase family 10	
AT3G56710	0.349386	0.507345	0.509489	1.221872	2.079116	1.273216	2.53262	2.22728	3.226282	11.28407	6.07051	8.110356	SIB1	3	21006638	21008068	-	1431	protein_coding	sigma factor binding protein 1 [Source:NCBI gene (formerly Entrezgene);Acc:824838] && sp|Q9LDH1|SIB1_ARATH Sigma factor binding protein 1, chloroplastic OS=Arabidopsis thaliana OX=3702 GN=SIB1 PE=1 SV=1 && PF05678:VQ motif	-
AT3G44300	4.76727	1.709971	3.245626	0.476036	1.955713	1.483691	18.39291	17.8065	18.59893	22.55892	32.23225	34.39824	NIT2	3	15983199	15985535	+	1535	protein_coding	nitrilase 2 [Source:NCBI gene (formerly Entrezgene);Acc:823555] && sp|P32962|NRL2_ARATH Nitrilase 2 OS=Arabidopsis thaliana OX=3702 GN=NIT2 PE=1 SV=1 && PF00795:Carbon-nitrogen hydrolase	TCP

AT1G09500	0.11769	0.23137	0.062928	0.378413	0.55523	0.235885	1.588096	2.688417	1.408687	0.65145	1.181211	1.213734	AT1G09500	1	3066674	3068796	+	1931	protein_coding	NAD(P)-binding Rossmann-fold superfamily protein [Source:NCBI gene (formerly Entrezgene);Acc:837475] && sp|A0A0B6VQ48|PAR1_ROSHC Phenylacetaldehyde reductase OS=Rosa hybrid cultivar OX=128735 GN=PAR PE=1 SV=1 && PF01370:NAD dependent epimerase/dehydratase family	NAC
AT3G07010	15.40777	13.21241	13.93623	17.36771	16.57835	16.22409	5.17307	4.167889	6.465177	6.562295	7.01836	7.224259	AT3G07010	3	2212289	2216483	-	3447	protein_coding	Pectin lyase-like superfamily protein [Source:NCBI gene (formerly Entrezgene);Acc:819886] && sp|Q9M8Z8|PLY8_ARATH Probable pectate lyase 8 OS=Arabidopsis thaliana OX=3702 GN=At3g07010 PE=2 SV=1 && PF00544:Pectate lyase	AP2

AT2G43510	10.18338	5.724127	7.451525	11.69392	15.49756	12.5362	49.5555	22.65448	35.02328	70.44881	74.2301	108.4441	TI1	2	18066960	18067860	+	761	protein_coding	trypsin inhibitor protein 1 [Source:NCBI gene (formerly Entrezgene);Acc:818952] && sp|Q42328|DF195_ARATH Defensin-like protein 195 OS=Arabidopsis thaliana OX=3702 GN=ATTI1 PE=1 SV=3 && -	-
AT2G14560	0	0.037532	0	0.175383	0.144106	0.022675	2.180121	0.594989	0.816204	4.8469 4	2.891806	0.8784 1	LURP1	2	6213617	6215932	+	2232	protein_coding	LURP-one-like protein (DUF567) [Source:NCBI gene (formerly Entrezgene);Acc:815943] && sp|Q9ZQR8|LURP1_ARATH Protein LURP1 OS=Arabidopsis thaliana OX=3702 GN=LURP1 PE=2 SV=1 && PF04525:LURP-one-related	bZIP
AT5G64750	0.554968	0.409135	0.395647	0.079661	0.212728	0.216283	1.3770 5	1.827883	1.736846	5.194333	3.259046	4.333801	ABR1	5	25891449	25893888	+	1638	protein_coding	Integrase-type DNA-binding superfamily protein [Source:NCBI gene (formerly Entrezgene);Acc:836596]	ERF

																				&& sp|Q9FGF8|ABR1_ARATH Ethylene-responsive transcription factor ABR1 OS=Arabidopsis thaliana OX=3702 GN=ABR1 PE=2 SV=1 && PF00847:AP2 domain	
AT4G30270	43.23567	36.80821	35.34926	48.37649	52.56635	50.2339	81.91737	110.5516	68.65919	253.6693	127.0407	121.0413	XTH24	4	14819192	14820595	-	1210	protein_coding	xyloglucan endotransglucosylase/hydrolase 24 [Source:NCBI gene (formerly Entrezgene);Acc:829150] && sp|P24806|XTH24_ARATH Xyloglucan endotransglucosylase/hydrolase protein 24 OS=Arabidopsis thaliana OX=3702 GN=XTH24 PE=1 SV=2 && PF00722:Glycosyl hydrolases family 16|PF06955:Xyloglucan endo-transglycosylase (XET) C-terminus	NAC

AT3G56400	1.545984	1.481653	2.011441	5.574459	5.050773	3.029734	5.206707	6.948059	3.700917	21.25368	12.52871	15.98966	WRKY70	3	20908711	20910623	-	1470	protein_coding	WRKY DNA-binding protein 70 [Source:NCBI gene (formerly Entrezgene);Acc:824807] && sp|Q9LY00|WRK70_ARATH Probable WRKY transcription factor 70 OS=Arabidopsis thaliana OX=3702 GN=WRKY70 PE=1 SV=1 && PF03106:WRKY DNA -binding domain	WRKY
AT3G56980	2.880492	0.758415	2.150231	0.128874	0.551517	1.08302	0.146014	0.039746	0.246476	2.051875	2.396551	2.72655	bHLH39	3	21086390	21087710	-	1215	protein_coding	basic helix-loop-helix (bHLH) DNA-binding superfamily protein [Source:NCBI gene (formerly Entrezgene);Acc:824865] && sp|Q9M1K0|ORG3_ARATH Transcription factor ORG3 OS=Arabidopsis thaliana OX=3702 GN=ORG3 PE=1 SV=1	bHLH

																			&& PF00010:Helix-loop-helix DNA-binding domain	
AT4G11280	6.704826	6.029439	7.436263	7.629639	11.58965	9.900258	11.09389	17.07366	18.13992	55.13197	30.01037	41.65082	ACS6	4	6863979	6866116	+	1871	protein_coding	1-aminocyclopropane-1-carboxylic acid (acc) synthase 6 [Source:NCBI gene (formerly Entrezgene);Acc:826730] && sp|Q9SAR0|1A16_ARATH 1-aminocyclopropane-1-carboxylate synthase 6 OS=Arabidopsis thaliana OX=3702 GN=ACS6 PE=1 SV=2 && PF00155:Aminotransferase class I and II	M-type_MADS
AT1G44350	4.584801	4.039473	3.697457	6.993311	6.914081	4.289199	8.271279	11.00499	7.133315	19.62293	15.90182	14.44449	ILL6	1	16834210	16838562	-	2295	protein_coding	IAA-amino acid hydrolase ILR1-like 6 [Source:NCBI gene (formerly Entrezgene);Acc:841026] && sp|Q8VYX0|ILL6_ARATH IAA-amino acid hydrolase ILR1-like 6 OS=Arabidopsis thaliana OX=3702 GN=ILL6 PE=1	C2H2

																			SV=2 && PF01546:Peptidase family M20/M25/M40	
AT2G27830	19.02517	14.26061	22.48178	30.64601	35.67685	27.027	4.99027	7.57858	9.367938	11.6982	11.79862	11.35403	AT2G27830	2	11860218	11861525	+	1308	protein_coding	- && - && -	C2H2
AT1G14250	0.302702	0.529287	0.166001	2.71398	1.73016	0.868569	2.88235	1.632806	0.84379	0.538988	0.335634	0.669608	AT1G14250	1	4868346	4871488	+	1952	protein_coding	GDA1/CD39 nucleoside phosphatase family protein [Source:NCBI gene (formerly Entrezgene);Acc:837986] && sp|Q6NQA8|APY5_ARATH Probable apyrase 5 OS=Arabidopsis thaliana OX=3702 GN=APY5 PE=2 SV=1 && PF01150:GDA1/CD39 (nucleoside phosphatase) family	FAR1
AT3G61028	0.095823	0.156984	0.227712	0.036679	0.056508	0	1.032991	0.712664	0.631347	0.385751	0.170521	0.205878	AT3G61028	3	22581473	22584938	-	1423	protein_coding	Putative endonuclease or glycosyl hydrolase [Source:NCBI gene (formerly Entrezgene);Acc:5008101]	C2H2

																				&& - && PF01936:NYN domain	
AT1G17147	4.468722	2.994946	3.215646	4.692519	6.087916	7.077997	0.639594	0.787085	1.653219	2.717682	3.674208	3.649325	AT1G17147	1	5863109	5864059	-	951	protein_coding	VQ motif-containing protein [Source:NCBI gene (formerly Entrezgene);Acc:6240987] && sp|Q1G3U8|VQ1_ARATH VQ motif-containing protein 1 OS=Arabidopsis thaliana OX=3702 GN=VQ1 PE=1 SV=1 && -	-
AT1G19660	51.13341	49.28824	45.22841	85.40705	70.54073	61.00841	32.00831	34.66713	34.64426	19.17923	29.17837	27.92634	AT1G19660	1	6799969	6802906	-	1852	protein_coding	Wound-responsive family protein [Source:NCBI gene (formerly Entrezgene);Acc:838553] && sp|Q93VH2|BBD2_ARATH Bifunctional nuclease 2 OS=Arabidopsis thaliana OX=3702 GN=BBD2 PE=2 SV=1 && PF02151:UvrB/uvrC motif|PF02577:Bifunctional nuclease	bHLH

AT5G24240	1.120425	0.367112	0.990144	1.44745	1.574747	1.985738	0.229074	0.119042	0.184553	0.826919	0.727753	0.944391	AT5G24240	5	8230916	8234150	-	2434	protein_coding	phosphatidylinositol 4-kinase gamma-like protein [Source:NCBI gene (formerly Entrezgene);Acc:832491] && sp|Q9FNF8|P4KG3_ARATH Phosphatidylinositol 4-kinase gamma 3 OS=Arabidopsis thaliana OX=3702 GN=PI4KG3 PE=2 SV=1 && PF00240:Ubiquitin family|PF00454:Phosphatidylinositol 3- and 4-kinase	bHLH
AT5G39850	12.33445	15.20904	13.42364	8.047199	11.9986	9.990801	28.99577	17.6976	27.40096	47.17176	28.69387	32.23416	AT5G39850	5	15949910	15951475	+	1041	protein_coding	Ribosomal protein S4 [Source:NCBI gene (formerly Entrezgene);Acc:833981] && sp|Q9FLF0|RS92_ARATH 40S ribosomal protein S9-2 OS=Arabidopsis thaliana OX=3702 GN=RPS9C PE=1 SV=1 && PF00163:Ribosomal protein	bHLH

																				S4/S9 N-terminal domain|PF01479:S4 domain	
AT4G27280	5.629712	7.241131	7.852544	9.816101	15.72249	11.76156	10.79268	14.36341	10.66873	41.04421	16.90715	20.50064	AT4G27280	4	13663415	13664621	-	1207	protein_coding	Calcium-binding EF-hand family protein [Source:NCBI gene (formerly Entrezgene);Acc:828836] && sp|O81831|CARP1_ARATH Calcium-binding protein KRP1 OS=Arabidopsis thaliana OX=3702 GN=KRP1 PE=2 SV=1 && PF13833:EF-hand domain pair	MYB_related
AT4G33985	5.829445	4.290324	5.247635	6.388326	6.805638	7.776067	11.02196	8.520176	8.05862	23.11007	10.67916	10.68101	AT4G33985	4	16288112	16288971	-	768	protein_coding	membrane insertase, putative (DUF1685) [Source:NCBI gene (formerly Entrezgene);Acc:829545] && - && PF07939:Protein of unknown function (DUF1685)	-
AT2G05540	15.38967	15.24947	19.36738	42.12257	38.384	20.66794	17.47007	71.26509	47.76114	14.9333	13.22211	13.20968	AT2G05540	2	2033154	2034283	+	824	protein_coding	Glycine-rich protein family [Source:NCBI gene (formerly	-

																				Entrezgene);Acc:815103] && - && PF07172:Glycine rich protein family	
AT1G70700	8.421108	15.76908	8.422023	27.71574	18.31734	13.1576	25.95905	25.68252	16.08527	36.83671	32.64138	29.367	TIFY7	1	26654529	26657350	+	1854	protein_coding	TIFY domain/Divergent CCT motif family protein [Source:NCBI gene (formerly Entrezgene);Acc:843407] && sp|Q8W4J8|TIF7_ARATH Protein TIFY 7 OS=Arabidopsis thaliana OX=3702 GN=TIFY7 PE=1 SV=2 && PF06200:tify domain|PF09425:Divergent CCT motif	-
AT1G04047	0.201561	0.247658	0.044905	0.925836	0.832045	0.336654	1.685847	0.642459	1.383355	5.832061	4.949861	6.39596	AT1G04047	1	270067	270517	+	451	lncRNA	ncRNA [Source:NCBI gene (formerly Entrezgene);Acc:28716041] && - && -	-
AT2G33830	232.9868	132.1811	202.1236	389.3559	304.2788	271.844	113.9683	106.1851	117.1737	38.58981	64.81531	69.23857	AT2G33830	2	14308754	14310608	-	1119	protein_coding	Dormancy/auxin associated family protein [Source:NCBI gene (formerly Entrezgene);Acc:817950]	-

																				&& sp|P93017|DRMH1_ARATH Dormancy-associated protein homolog 1 OS=Arabidopsis thaliana OX=3702 GN=DRMH1 PE=1 SV=1 && PF05564:Dormancy/auxin associated protein	
AT2G42065	0.172384	0.070603	0.15362	0	0	0	0.320403	0.76314	1.54593	5.522778	2.484793	6.011437	AT2G42065	2	17551475	17553028	-	791	protein_coding	DnaJ domain protein [Source:NCBI gene (formerly Entrezgene);Acc:28718346] && sp|P25686|DNJB2_HUMAN DnaJ homolog subfamily B member 2 OS=Homo sapiens OX=9606 GN=DNAJB2 PE=1 SV=3 && PF00226:DnaJ domain	-
AT2G15960	23.61024	22.05628	23.11533	40.3471	36.78127	26.97905	31.98394	28.40491	33.1772	15.27755	16.36555	15.90072	AT2G15960	2	6947096	6947866	+	771	protein_coding	stress-induced protein [Source:NCBI gene (formerly Entrezgene);Acc:816091] && - && -	-

AT3G16860	1.31221	1.074878	1.25411	1.561452	1.659813	1.609366	1.887538	2.950023	1.795977	6.698859	3.979879	2.989054	COBL8	3	5759375	5762264	-	2390	protein_coding	COBRA-like protein 8 [Source:NCBI gene (formerly Entrezgene);Acc:820941] && sp|Q9LIB6|COBL8_ARATH COBRA-like protein 8 OS=Arabidopsis thaliana OX=3702 GN=COBL8 PE=2 SV=1 && PF04833:COBRA-like protein	M-type_MADS
AT3G49620	2.585854	1.81557	2.357048	4.27597	4.130356	2.550261	0.626182	0.612278	1.557705	25.80144	20.5417	11.92725	DIN11	3	18393747	18397050	+	1538	protein_coding	2-oxoglutarate (2OG) and Fe(II)-dependent oxygenase superfamily protein [Source:NCBI gene (formerly Entrezgene);Acc:824124] && sp|Q8H113|DIN11_ARATH Probable 2-oxoglutarate-dependent dioxygenase DIN11 OS=Arabidopsis thaliana OX=3702 GN=DIN11 PE=2 SV=1 && PF03171:2OG-Fe(II)	Dof

																				oxygenase superfamily|PF14226:non-haem dioxygenase in morphine synthesis N-terminal	
AT1G07373	1.218084	1.41114	1.023464	0.159859	0.656753	0.077504	0.543361	0.33279	0.38217	1.15585	1.449216	0.966312	AT1G07373	1	17392374	17393026	-	653	lncRNA	ncRNA [Source:NCBI gene (formerly Entrezgene);Acc:28716644] && - && -	-
AT2G27385	35.30397	38.19472	32.10204	87.77876	57.91809	38.44481	51.18645	42.23955	44.98008	21.79573	27.69001	30.47823	AT2G27385	2	11716001	11717437	-	1169	protein_coding	Pollen Ole e 1 allergen and extensin family protein [Source:NCBI gene (formerly Entrezgene);Acc:817283] && - && -	-
AT1G74930	0.376258	0.809041	0.989138	3.132503	3.195148	2.409019	1.174883	3.497934	1.322158	9.769751	8.918623	6.8652	ORA47	1	28143851	28145058	+	1208	protein_coding	Integrase-type DNA-binding superfamily protein [Source:NCBI gene (formerly Entrezgene);Acc:843832] && sp|Q9S7L5|ERF18_ARATH Ethylene-responsive transcription factor ERF018 OS=Arabidopsis thaliana OX=3702 GN=ERF018	ERF

																			PE=2 SV=1 && PF00847:AP2 domain	
AT3G04485	2.847216	2.894118	2.929416	4.725993	4.579227	3.516208	4.849403	4.675143	3.638193	2.474741	3.288775	2.926056	AT3G04485	3	1198114	1200104	+	878	ncRNA	ncRNA [Source:NCBI gene (formerly Entrezgene);Acc:5007982] && sp|Q9M8X2|U1148_ARATH U11/U12 small nuclear ribonucleoprotein 48 kDa protein OS=Arabidopsis thaliana OX=3702 GN=SNRNP48 PE=3 SV=1 && -	-
AT3G47960	20.11657	23.12665	18.13413	27.9901	26.92303	23.89745	23.5731	27.51991	29.10946	39.25973	41.28412	36.75727	GTR1	3	17697942	17700923	-	2247	protein_coding	Major facilitator superfamily protein [Source:NCBI gene (formerly Entrezgene);Acc:823951] && sp|Q944G5|PTR44_ARATH Protein NRT1/ PTR FAMILY 2.10 OS=Arabidopsis thaliana OX=3702 GN=NPF2.10 PE=1 SV=3 && PF00854:POT family	BBR-BPC

AT2G15890	53.34015	76.45383	83.85551	142.7451	110.0077	85.83788	51.07844	48.33614	54.4041	27.34184	33.70483	35.16524	MEE14	2	6920369	6922161	-	1622	protein_coding	maternal effect embryo arrest 14 [Source:NCBI gene (formerly Entrezgene);Acc:816085] && sp|Q9XIM0|MEE14_ARATH CCG-binding protein 1 OS=Arabidopsis thaliana OX=3702 GN=MEE14 PE=1 SV=1 && -	-
AT2G04066	0.088085	0	0.039248	0.404605	0.467507	0.098082	0.392929	0.23397	0.435274	1.640034	0.658355	0.829805	AT2G04066	2	1352887	1353517	-	516	protein_coding	MATE efflux family protein [Source:NCBI gene (formerly Entrezgene);Acc:814942] && sp|Q8GXM8|DTX2_ARATH Protein DETOXIFICATION 2 OS=Arabidopsis thaliana OX=3702 GN=DTX2 PE=2 SV=1 && PF01554:MatE	HD-ZIP
AT2G01300	0.968647	0.976558	0.996009	1.654236	2.548553	1.576388	1.938878	2.269436	1.118235	5.574225	3.261863	3.398837	AT2G01300	2	151057	151971	-	915	protein_coding	mediator of RNA polymerase II transcription subunit [Source:NCBI gene (formerly	-

																			Entrezgene);Acc:814658] && - && -	
AT2G39700	53.60296	27.64423	39.49175	28.6197	25.35634	26.50295	26.69558	24.14574	31.24457	29.4589	43.56391	38.95683	EXPA4	2	16543832	16545746	-	1500	protein_coding	expansin A4 [Source:NCBI gene (formerly Entrezgene);Acc:818553] && sp|O48818|EXPA4_ARATH Expansin-A4 OS=Arabidopsis thaliana OX=3702 GN=EXPA4 PE=1 SV=1 && PF03330:Lytic transglycolase|PF01357:Pollen allergen	bZIP
AT1G69252	37.08895	35.01107	36.27225	45.17094	42.18652	41.22086	32.68098	26.73014	28.61895	22.40552	24.0672	24.9938	AT1G69252	1	26036570	26037659	+	981	ncRNA	ncRNA [Source:NCBI gene (formerly Entrezgene);Acc:6240760] && - && -	-
